# The gut microbiota composition affects dietary polyphenols-mediated cognitive resilience in mice by modulating the bioavailability of phenolic acids

**DOI:** 10.1038/s41598-019-39994-6

**Published:** 2019-03-05

**Authors:** Tal Frolinger, Steven Sims, Chad Smith, Jun Wang, Haoxiang Cheng, Jeremiah Faith, Lap Ho, Ke Hao, Giulio M. Pasinetti

**Affiliations:** 1 Department of Neurology, Icahn School of Medicine at Mount Sinai, New York, 10029 New York USA; 2 Geriatric Research, Education and Clinical Center, James J. Peters Veterans Affairs Medical Center, Bronx, New York, 10468 New York USA; 3 Department of Genetics and Genomic Sciences, Icahn School of Medicine at Mount Sinai, New York, 10029 New York USA; 4 Institute of Genomics and Multiscale Biology, Icahn School of Medicine at Mount Sinai, New York, 10029 New York USA

## Abstract

Dietary polyphenols promote memory in models of sleep deprivation (SD), stress, and neurodegeneration. The biological properties of dietary polyphenols greatly depend upon the bioavailability of their phenolic metabolites derivatives, which are modulated by gut microbiota. We recently demonstrated that supplementation with grape-derived bioactive dietary polyphenol preparation (BDPP) improves SD-induced cognitive impairment. This study examined the role of the gut microbiota in the ability of BDPP to prevent memory impairment in response to SD. C57BL6/J mice, treated with antibiotics mix (ABX) or BDPP or both, were sleep-deprived at the end of a fear conditioning training session and fear memory was assessed the next day. Gut microbiota composition was analyzed in fecal samples and BDPP-driven phenolic acid metabolites extraction was measured in plasma. We report that the beneficial effect of BDPP on memory in SD is attenuated by ABX-induced dysbiosis. We identified specific communities of fecal microbiota that are associated with the bioavailability of BDPP-derived phenolic acids, which in turn, are associated with memory promotion. These results suggest the gut microbiota composition significantly affects the bioavailability of phenolic acids that drive the dietary polyphenols’ cognitive resilience property. Our findings provide a preclinical model with which to test the causal association of gut microbiota-polyphenols, with the ultimate goal of potential developing dietary polyphenols for the prevention/treatment of cognitive impairment.

## Introduction

Dietary plant-derived polyphenols possess diverse biological activities associated with physiological resilience to cognitive deficits, including modulations in synaptic plasticity, inflammatory pathways, and promotion of neurogenesis^1–4^. There is a growing interest in the development of polyphenolic compounds for attenuating cognitive deterioration and preventing the occurrence of detrimental neuropathology in the brain. The biological properties of dietary polyphenols greatly depend upon their bioavailability which, in turn, is largely influenced by the gut microbiota. While only 5–10% of the total dietary polyphenolic intake is absorbed in the small intestine, the remaining 90–95% accumulates in the large intestinal lumen, where it is then subjected to the enzymatic activities of the gut microbial community^5–10^. Studies showed that catechin ((+)-C) and epicatechin ((−)-EC), major components of grape seed polyphenol extract, are metabolized by gut microbiota fermentation^11,12^ and gut microbiota fermentation products of C and EC have been identified in urine and plasma^13,14^. The gut microbiota is therefore responsible for the breakdown of polyphenols into low-molecular-weight phenolic metabolites– modified polyphenol conjugates and phenolic acids that can be more efficiently absorbed^15,16^.

Studies from our laboratory and others have shown that phenolic metabolites derived from multiple dietary sources, including a specific grape-derived bioactive dietary polyphenol preparation (BDPP) comprised of Grape Seed Polyphenol Extract (GSPE), Concord Grape Juice (CGJ), and resveratrol (RSV), are effective in protecting against hippocampus-dependent memory impairment under conditions of sleep deprivation (SD) and stress^17–19^. Further, they exhibit beneficial biological activities in neurodegenerative disorders such as Alzheimer’s disease and tauopathies^1,2,20–22^. We recently identified specific bioavailable phenolic metabolites that were able to restore SD-induced impaired cognitive function^17^. Taken together, these finding suggest that the gut microbiota plays a key role in promoting the cognitive resilience effects mediated by polyphenol-rich dietary consumption.

This study was designed to examine the role of the gut microbiota in promoting BDPP cognitive resilience in a mouse model of SD. Mice receiving ABX, BDPP or ABX supplemented with BDPP were tested in the fear conditioning paradigm.

Our studies were designed to explore the role of gut microbiota in BDPP-mediated cognitive resilience, hypothesizing the composition of gut microbiota affect polyphenols bioavailability in association with their cognitive resilience effect. We utilized the SD-induced memory impairment model to test the effect of ABX-induced dysbiosis on memory function in sleep-deprived mice treated with BDPP. We explored the potential effect of ABX on microbiota conversion of dietary polyphenols into phenolic acids in circulating blood in association with their effect on memory function. Our studies suggest a critical role for gut microbiota composition in the efficacy of dietary polyphenols to attenuate cognitive dysfunction and provide a preclinical model that tests their causal association.

## Results

### Gut microbiota contributes to dietary polyphenol-mediated promotion of resilience

**Figure 1:**
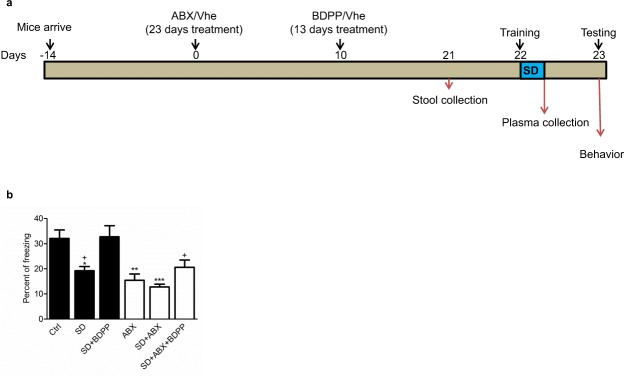
ABX treatment impairs cognitive function and reverse BDPP memory promoting effect. C57BL6 mice were randomly grouped into 6 groups: non sleep-deprived vehicle treated (Ctrl), sleep-deprived vehicle treated (SD), sleep-deprived BDPP treated (SD + BDPP), non sleep-deprived ABX treated (ABX), sleep-deprived ABX treated (SD + ABX), sleep-deprived treated with ABX in addition to BDPP (SD + ABX + BDPP). (**a**) Treatment scheme. Mice were sleep deprived for 5 h immediately after a fear conditioning training trial and tested for memory performance the next day. (**b**) Freezing percent across testing trial is significantly affected by sleep deprivation, BDPP and ABX treatment (1-way ANOVA, F(5,58) = 7.861, p < 0.0001). Sleep-deprived mice show decrease in freezing compared to both non sleep-deprived mice and sleep-deprived BDPP-treated mice (Tukey’s Multiple Comparison Test, *P < 0.05 Ctrl, *n* = 9 vs. SD, *n* = 10 and SD, *n* = 10 vs. SD + BDPP, n = 10). ABX treated mice showed reduced freezing compare to vehicle treated mice (Tukey’s Multiple Comparison Test, **P < 0.005 ABX, *n* = 9 vs. Ctrl, *n* = 9). ABX treatment reverse BDPP-mediated increase in freezing in sleep-deprived mice. (Tukey’s Multiple Comparison Test, *P < 0.05 SD + ABX + BDPP, *n* = 12 vs. SD + BDPP, *n* = 10). Data are means + SEM.

To examine the role of gut microbiota in modulating the effect of BDPP treatment on promotion of cognitive resilience, male C57BL6 mice were randomly grouped into 6 treatment groups receiving either vehicle (Ctrl, SD) or antibiotic mix (ABX, SD + ABX) for 23 (Days 10–23) days or supplemented with BDPP (SD + BDPP, SD + ABX + BDPP) for 13 days (Days 10–23). On Day 22, we performed behavioral training for fear conditioning. At the end of the training session, Mice in Ctrl and ABX groups were left undisturbed in their home cages and mice in SD, SD + BDPP, SD + ABX, SD + ABX + BDPP were kept awake for 5 h. The next day (Day 23), mice were tested for memory performance (Fig. 1a). We did not find that any treatment had an effect on food intake or liquid consumption. In accordance to previous report^23^, mice in ABX treatment groups showed a small reduction of ~5% in body weight (Supplementary Fig. S1).

The learning paradigm for contextual fear conditioning is used to assess memory consolidation of fearful experiences^24^. We found that in vehicle-treated mice, SD led to significantly impaired contextual memory compared to non-sleep deprived mice (Fig. 1b, Freezing behavior: SD, 19.20% ± 1.69 vs. Ctrl, 32.11% ± 3.32, *p < 0.05). Treatment with BDPP restored the SD-induced contextual memory impairment (Fig. 1b, Freezing behavior: SD + BDPP, 32.70 ± 4.46 vs. SD, 19.20 ± 1.69, + p < 0.05). Interestingly, in comparison to vehicle-treated mice, ABX treatment itself significantly impaired contextual memory in non-sleep deprived mice (Fig. 1b, freezing behavior: ABX, 15.44 ± 2.50 vs. Ctrl, 32.11 ± 3.32, **p < 0.005) and induce even further impairment in sleep deprived mice (Fig. 1b, freezing behavior: ABX + SD, 12.78 ± 1.11 vs. Ctrl, 32.11 ± 3.32, ***p < 0.0005), suggesting that the gut microbiome is necessary for cognitive function under normal and sleep deprivation conditions. Importantly, we found that ABX treatment prevented the beneficial effect of BDPP on contextual memory in sleep-deprived mice (Fig. 1b, Freezing behavior: SD + ABX + BDPP, 20.58 ± 2.90 vs. SD + BDPP, 32.70 ± 4.46, p < 0.05). These results suggest gut microbiota dysbiosis reduces the efficacy of dietary polyphenols in promoting resilience against sleep deprivation-mediated cognitive impairment in mice. Neither ABX mix nor BDPP treatment affected mice basal freezing behavior, ranging from 0–5% percent of freezing or the mice general motor function (Supplementary Fig S2). Therefore, the differences among groups in freezing behavior on testing day can be contributed to memory consolidation only.

### ABX treatment reduces BDPP memory promoting effect by reducing bioavailability of specific phenolic acids

**Figure 2:**
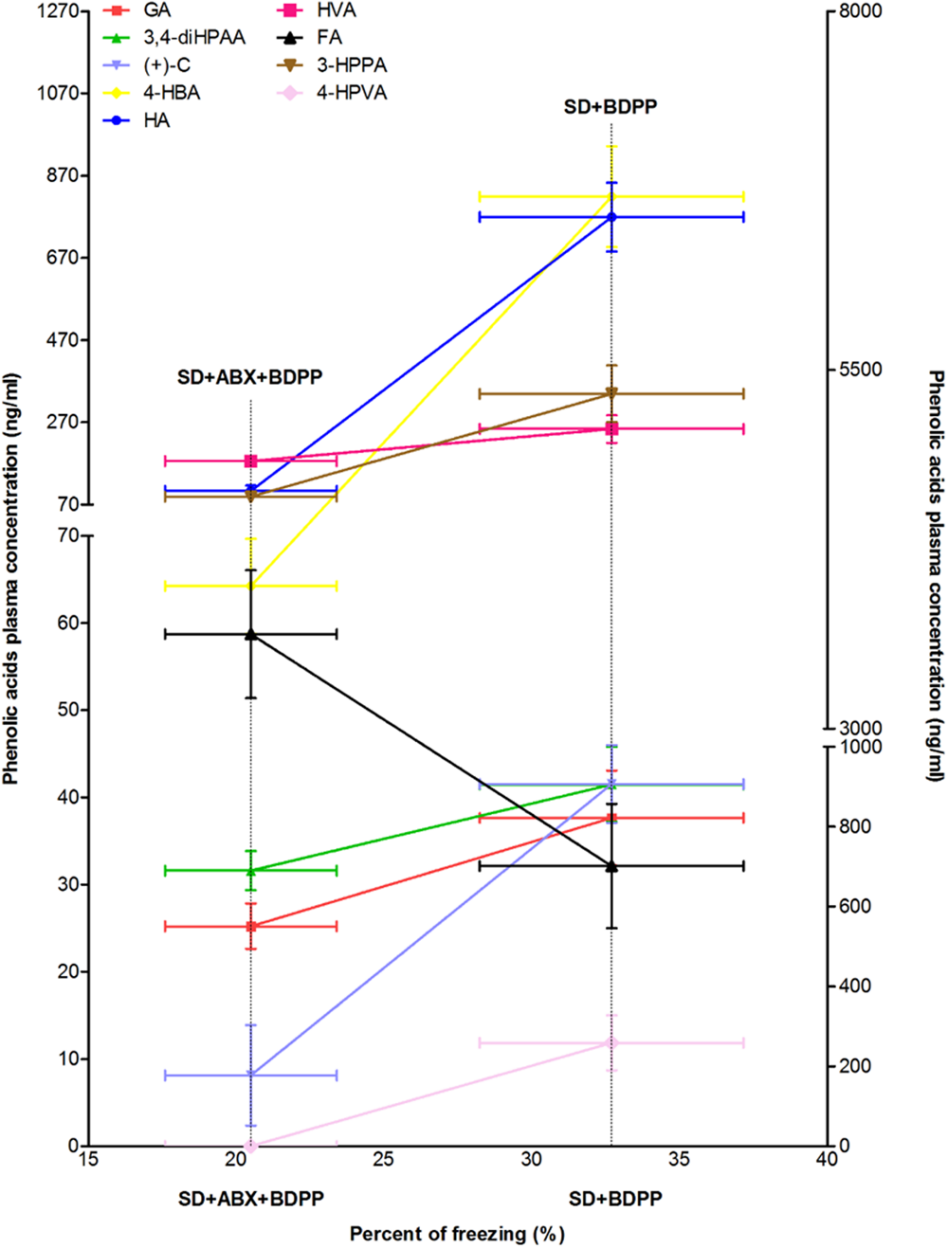
Reduced plasma concentration of specific BDPP-driven phenolic acids following ABX treatment associates with attenuation of BDPP memory promoting effect. A scatterplot of group mean plasma phenolic acid concentrations collected on Day 22 and freezing percent in fear conditioning test conducted on Day 23 in SD + ABX + BDPP, *n* = 10 vs. SD + BDPP, *n* = 10 treated C57BL6 mice. Data are means + SEM.

We first investigated the effect of ABX treatment on phenolic acid bioavailability. Comparing plasma concentration of the phenolic acids in the SD + BDPP and SD + ABX + BDPP treatment groups, we found that out of 16 phenolic acids (e.g; Caffeic acid (CA), Trans-p-coumaric acid (p-CA), 5-(4-Hydroxyphenyl) valeric acid (4-HPVA), Hippuric acid (HA), 3-Hydroxyphenylacetic acid (3-HBA), 4-Hydroxybenzoic acid (4-HBA), 3-(3,4-Dihydroxyphenyl)propionic acid (3,4 di-HPPA), 3-(3-Hydroxyphenyl)propionic acid (3-HPPA), (+)-C, Dihydrocoumaric acid (di-HCA), Homovanillic acid (HVA), 3, 4-Dihydroxyphenylacetic acid (3, 4-diHPAA), 3,4-Dihydroxybenzoic acid (3,4-diHBA). Ferulic acid (FA), Vanillic acid (VA) and Gallic acid (GA) treatment with ABX significantly affected the concentration of 9 out of 16 phenolic acids in SD + BDPP mice **(**Phenolic acids concentration in each group and comparison of SD + BDPP vs. SD + ABX + BDPP are summarized in Supplementary Table S1). Specifically, group SD + ABX + BDPP showed 2–4-fold reduction in plasma concentration of the following phenolic acids: HA, 4-HBA, 3-HPPA, ( + )-C, HVA, 3, 4-diHPAA, and GA and 1.8-fold increase in concentration of FA when compared to SD + BDPP group. Interestingly, the phenolic acid 4-HPVA plasma concentration found in BDPP sleep-deprived mice (SD + BDPP; 200 ng/ml) was completely eliminated by ABX treatment (SD + ABX + BDPP; 0 ng/ml). The ABX-induced changes in the bioavailability of phenolic acids correlated with 37% reduction of freezing behavior in SD + ABX + BDPP compared with SD + BDPP (Fig. 2). These findings suggest a role of ABX-induced dysbiosis in BDPP’s memory promoting effect in SD by modulating the bioavailability of BDPP-derived phenolic acids.

### Both ABX and BDPP-treated mice display alterations in gastrointestinal microbiome composition

**Figure 3:**
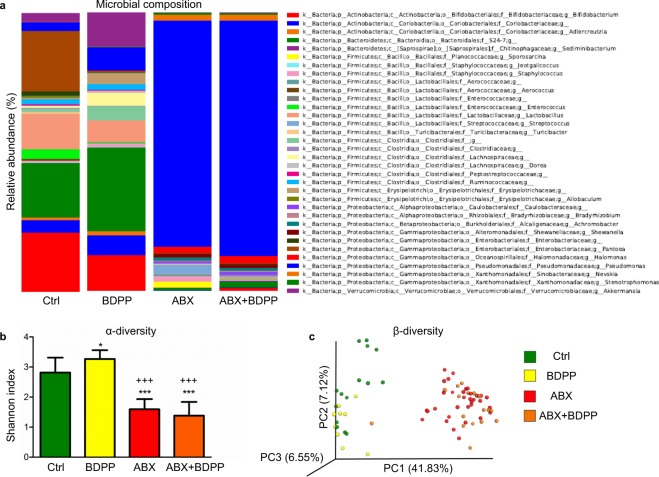
Gut OTUs composition is affected by both ABX and by BDPP treatment. Illumina MiSeq based sequencing of the 16 s rRNA gene from fecal contents of male C57BL6 mice, was performed in stool collected on day 21. Microbial diversity histogram (**a**, left panel) and phylogenetic classification (**a**, right panel) were generated based on quality-controlled OTU reads. Only families and genus’ with relative abundance >0.5% were included. (**b**) α-diversity analysis of the sequencing data using the Shannon index. Microbial diversification is significantly affected by both BDPP and ABX treatment (1-way ANOVA, F (3,73) = 75.11, p < 0.0001). ABX treatment decrease microbial diversification in both vehicle and BDPP-treated C57BL6 mice (Tukey’s multiple comparisons test, ***P < 0.0005; ABX, *n* = 33 and ABX + BDPP, *n* = 17 vs. Ctrl, *n* = 15 and ^+++^P < 0.0005; ABX, *n* = 33 and ABX + BDPP, *n* = 17 vs. BDPP, *n* = 9). BDPP treatment increased microbial diversification compare to vehicle treated mice (Tukey’s multiple comparisons test, *P < 0.05 BDPP, *n* = 9 vs. Ctrl*, n* = 15). (**c**) Principal co-ordinate analysis of weighted β-diversity, accounting for both presence and relative abundance of OTUs, demonstrates distinct alterations in microbial diversity in ABX-treated groups (ABX, *n* = 33; ABX + BDPP, *n* = 17) and in BDPP treated mice (BDPP, *n* = 9) compared to vehicle treated mice (Ctrl, *n* = 15). The percentage of data variance explained by each PC is displayed. Data are displayed as X/Y scatter or mean + SEM.

To characterize the effect of ABX and of BDPP treatment on the gut microbial composition, we employed Illumina® MiSeq-based high throughput amplicon sequencing of the 16S rRNA microbial gene to identify microbial communities in fecal contents of male C57BL6 mice treated with vehicle (Ctrl), ABX (ABX), BDPP (BDPP), or a combination of ABX + BDPP (ABX + BDPP). The food, water, cages, bedding and all other animal facility conditions were kept the same for all mice. This was shown enough to induce a baseline composition of fecal microbiota that is uniform^25^. Feces were then collected on treatment day 21, prior to SD procedure. Using the QIIME for taxonomic classification analysis of operational taxonomic units (OTUs) derived from the primary sequencing, we identified 34 genera and 62 species of OTUs in mice fecal samples. Comparison of the percentage of OTUs genus among all treatment groups revealed ABX induced similar dramatic alternation of microbial composition in vehicle treated mice (Fig. 3a; ABX vs. Ctrl, Supplementary Table S2) or BDPP treated mice (Fig. 3a; ABX + BDPP vs. BDPP, Supplementary Table S2) as compared to their respective controls. Interestingly we observed an ABX-induced expansion of the genus *Pseudomonas* in vehicle treated mice (81.4% in ABX vs. 3.1% in Ctrl) or in BDPP treated mice (Fig. 3a, Supplementary Table S2, 84.7% in ABX + BDPP vs. 8.4% in BDPP) and an extinction of the species Pantoea in BDPP treated compare to vehicle treated mice (Fig. 3a, Supplementary Table S2, 0.4% in BDPP vs. 21.7% in Ctrl). Comparison of the quantified OTUs among all treatment groups reveled ABX treatment significantly altered all identified 62 OTUs, (ABX vs. Ctrl, Supplementary Table S3) and 48 OTUs in BDPP treated mice (ABX + BDPP vs. BDPP, Supplementary Table S3) as compared to their respective controls. Significant alterations in OTUs composition were observed in feces contents of BDPP-treated non-sleep deprived mice (BDPP) compared to non-sleep deprived vehicle-treated controls (Ctrl) where 30 out of 62 measured OTUs species were different (BDPP vs Ctrl, Supplementary Table S3). Results suggest that in the context of ABX treatment, the BDPP’s impact on the microbiome is abolished.

We then assessed the gut microbial diversity within each treatment group (α-diversity) by calculating Shannon index at 6092 sequences/sample (Supplementary Table S4), which assumes that all microbial species are represented in our samples and that random sampling occurs. Using this index, we confirm a significant ABX-induced decrease in microbial α-diversity in vehicle treated mice (Fig. 3b; ABX, 1.59 ± 0.05 vs. Ctrl, 2.81 ± 0.13, p < 0.0005) or BDPP treated mice (Fig. 3b; ABX + BDPP, 1.38 ± 0.11 vs. BDPP, 3.27 ± 0.09, p < 0.0005). Interestingly, we found an increase in microbial α-diversity in mice that were treated with BDPP compare to vehicle-treated mice (Fig. 3b; BDPP, 3.27 ± 0.09 vs. Ctrl, 2.81 ± 0.13, p < 0.05). We then assessed the dissimilarity of individual microbial communities between treatment groups (β-diversity). PCA plotting of weighted β-diversity PCA dissimilarity matrix data, accounting for presence and abundance of OTUs, revealed a distinct clustering effect in C57BL6 mice treated with either ABX (ABX) or ABX supplemented with BDPP (ABX + BDPP) compared to vehicle-treated (Ctrl) and BDPP-treated (BDPP) mice and clustering effect in BDPP compared to Ctrl (Fig. 3c). Collectively these data highlight that microbial diversity is altered by both ABX and BDPP treatment and suggest that, in the context of ABX treatment, the BDPP’s impact on the microbiome is abolished.

### ABX treatment in the context of BDPP affects OTUs association to freezing behavior via modulating the bioavailability of memory-promoting phenolic acids

**Figure 4:**
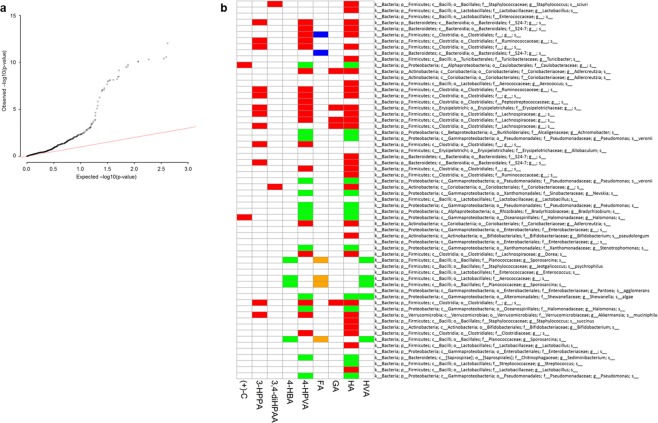
ABX treatment in the context of BDPP effects OTUs association with phenolic acids plasma concentration. (**a**) Quantitle-quantile plot comparing the observed p-value of fecal OTUs proportion collected on Day 21 (x-axis) vs. BDPP-driven phenolic acid plasma concentration collected on Day 22 (y-axis) against expected p-value under null hypothesis (i.e., no associations) in SD + BDPP, *n* = 9 vs. SD + ABX + BDPP, *n* = 17) mice groups by plotting their quantiles against each other. Points that lie approximately on the line y = x represent similar distributions. Points above the line y = x represent observed p-values that are more significant than expected. (**b**) Heat map visualizing the inferred influence of the specific OTUs on behavior traits through modifying plasma phenolic acids’ concentration. Red, OTUs positively associate with freezing percent through positive association with the particular phenolic acid; Orange, OTUs negatively associate with freezing percent through negative association with the particular phenolic acid; Blue, OTUs positively associate with freezing percent through negative association with the particular phenolic acid; Green, OTUs negatively associate with freezing percent through positive association with the particular phenolic acid; White, no significant influence of OTU on freezing percent detected.

Investigating OTUs association with freezing behavior revealed that the ABX-induced decrease in freezing behavior (Fig. 1b. ABX vs. Ctrl) was associated with alternation in 17 OTUs. The most significant finding included a positive association with freezing percent of the genus Lactobacillus and negative association of genera Nevskia, Halomonas, veronii and Pseudomonas (Supplementary Table S5, comparing ABX vs. Ctrl). However, no OTUs were associated with the BDPP-memory promoting effect, shown as BDPP-induced increase of freezing behavior in sleep deprived mice (Fig. 1b, Supplementary Table S5, comparing SD + BDPP vs. SD) or with the ABX-induced masking of BDPP-memory promoting effect, shown as decrease of freezing behavior in ABX and BDPP-treated compared to BDPP-treated, sleep deprived mice (Fig. 1b, Supplementary Table S5, comparing ABX + SD + BDPP vs. SD + BDPP). Together these results suggest that while ABX-induced modulation of gut microbiota might directly interfere with normal memory consolidation, it does not directly interfere with the beneficial effect of BDPP on memory consolidation, raising the possibility for an indirect effect of ABX on extending BDPP-induced memory promotion. We therefore asked whether antibiotic treatment in the context of BDPP would modulate the association between OTUs and the bioavailability of BDPP-derived phenolic acids that were found to be specifically associated with memory promotion. Focusing on groups ABX + SD + BDPP vs. SD + BDPP, we investigated the association between OTUs and the 9 phenolic acids that exhibited plasma concentrations in association with freezing behavior in these two groups (Fig. 2). The overall association (Wald’s test) between OTUs and phenolic acids bioavailability were detected and visualized in a quantile-quantile plot (Fig. 4a). Integration of the association between phenolic acid and freezing percent (Fig. 2), and the association between phenolic acid and OTUs (Supplementary Table S6) indicates the influence of OTUs on behavior traits through modification of the concentration of phenolic acids in the plasma (Fig. 4b). These results demonstrate the predicted effect of a given OTU on freezing percent, suggesting that their ability to modulate the bioavailability of BDPP-derived phenolic acids in plasma may be a possible mechanism. Our complete analyses identified 110 significant phenolic acids - OTU pair-wise significant correlations (Supplementary Table S6), where the most significant included the negative correlation between HA and the veronii species (Supplemental Table 6, ABX + SD + BDPP vs. SD + BDPP, FDR = 2.2e-9) and the positive correlation between 4-HPVA and the Dorea genus (Supplementary Table S6, ABX + SD + BDPP vs. SD + BDPP, FDR = 3.6e-9). We found 52 OTUs associated with at least one or more phenolic acids, including the Adlercreutzia genus that was positively associated with three phenolic acids; HA, 4-HPVA, and GA (Supplementary Table S6, ABX + SD + BDPP vs. SD + BDPP). Interestingly when an OTU is associated with changes multiple phenolic acids, the direction of their association is mostly consistent, and it is relatively rare that an OTU modulates multiple phenolic acids in different directions.

## Discussion

The present study shows that ABX treatment attenuates dietary polyphenols’ memory promoting effect in a model of SD. This behavioral change is associated with distinct alterations in the profile of circulating dietary polyphenol-driven phenolic acids, mediated by ABX-induced dysbiosis of the gut microbial community. Our observations suggest a role for gut microbiota in dietary polyphenols’ efficacy in promoting cognitive resilience.

In line with previously reported data^17^, we show that treatment with dietary polyphenols is effective in protecting against impaired memory under conditions of SD. Memory promotion was accompanied by dietary polyphenol-induced modification of 74.5% of the gut microbiota composition. Such an effect on gut microbiota is supported by studies of dietary polyphenol-rich extracts administration in both animals^26–29^ and humans^30,31^. Interestingly, ABX-mediated gut microbiota dysbiosis both attenuated the dietary polyphenols’ memory promoting effect and eliminated their effect on gut microbial composition. These findings suggest causal association of the bioavailability of dietary polyphenols that mediate cognitive resilience with gut microbiota composition following the completion of both the treatment regimen and the behavioral tasking. To further investigate such a causal association, future studies will analyze the change in phenolic acids plasma concentration and OTUs composition and their association to the behavioral outcome before and after the administration of BDPP and ABX.

Supporting other studies exploring the effect of antibiotic drugs on memory performance^32–35^, we found that ABX treatment itself resulted in gut microbiota dysbiosis and induced memory impairment in non sleep-deprived mice. We found 27.4% of ABX-induced gut microbiota depletion to be significantly associated with memory impairment. However, the change in microbiota composition seen following dietary polyphenols treatment in the absence or presence of ABX was not associated with cognitive performance. While this could be a result of low sample size of dietary polyphenol-treated mice, another explanation may stem from the evidence showing that specific phenolic metabolites derived from BDPP exert the beneficial biological activities on cognitive performance^17–19^. We recently reported that a BDPP-driven phenolic acid, 3-HPPA, which was found to accumulate in the brain, potentially interferes with the assembly of neurotoxic β-amyloid aggregates, which play key roles in neurodegeneration^36^. Here, we report an association between ABX-treatment induced alternation in plasma concentration of BDPP-driven phenolic acids, including 3-HPPA, and cognitive performance. We found that specific microbiota species are associated with BDPP-driven phenolic acid plasma concentrations. The most significant findings pointed out that ABX induced lower proportions of the *veronii* genus and higher proportions of the phenolic acid HA. In metabolomics analyses, plasma concentration of HA was found to be higher in humans with mild cognitive impairment when compared to control subjects^37^. ABX-induced lowered proportions of the Adlercreutzia genus was associated with lower proportions of the phenolic acid GA. GA was found to improve memory deficit and hippocampal long-term potentiation following oral administration in rats^38^. Together, these findings suggest that the association of dietary polyphenols-mediated gut microbiota composition with cognitive performance is mediated by its significant effect on phenolic acid concentration, which drives the cognitive response. The observed extensive association between microbiota species and phenolic acids suggest a functional mechanism by which the gut microbiome modifies the bioavailability of dietary polyphenols-driven phenolic acid plasma concentration, which in turn influences cognitive resilience. In order to become bioactive in the human body, polyphenols must undergo diverse intestinal transformations, modifications that are the result of the action of microbiota metabolism on backbone reorganizations. Our findings shed light on the importance of specific microbial taxa in the recognition of polyphenol structure and subsequent generation of compounds that are absorbed and that confer cognitive resilience. Data on the impact of gut microbiota on polyphenol metabolites and their mechanisms of action to promote cognitive function are scarce. Studies exploring the gut microbiota-brain axis indicated dysbiosis mediated cognitive deficits were accompanied by reduced expression of hippocampal *c-Fos*^39,40^, an immediate early gene that is a target of the cAMP response elements-binding protein, CREB, which is required for hippocampus-dependent long-term memory formation^41^. We previously showed that dietary polyphenols promote memory under stressful conditions and models of neurodegeneration through activation of CREB^17,18,21,42^. We recently identified specific BDPP-driven phenolic metabolites that were able to restore sleep deprivation-induced impaired memory through upregulation of CREB signaling^17^. Modulation of CREB signaling implies that *c-Fos* signaling may be an important event through which dietary polyphenols promote memory function^43^, suggesting a mechanism of action for gut microbiota-derived polyphenol metabolites in inducing cognitive resilience.

Our findings suggest that the efficacy of dietary polyphenols in promoting resilience against sleep deprivation-mediated cognitive impairment may be causally attributable to the composition of the gut microbiota. Given the safety and tolerability of BDPP, our studies provide preclinical evidence to support the hypothesis that not only the food polyphenols, but also gut microbiota composition must be taken into account for the potential implementation of polyphenols for the prevention/treatment of cognitive dysfunction. The results also support the potential translational application of prebiotics to enhance dietary polyphenol compounds – mediated resilience to cognitive deficits.

## Materials and Methods

### Chemicals

Polyphenol-free diet (AIN-93G) was purchased from Research Diets, Inc. (New Brunswick, NJ). Food-grade resveratrol was purchased from ChromaDex (Irvine, CA, USA). GSPE was purchased from Supplement Warehouse (UPC 603573579173, Bolingbrook, IL, USA). One lot of the resveratrol and one lot of the GSPE were used for this particular study and were stored at 4 °C in the dark. Concord purple grape juice was purchased from Welch Foods Inc. (Concord, MA, USA). The ABX were purchased as follows; Ampicillin (Sigma-Aldrich, MO, USA; A9518), Vancomycin (Amresco Inc. OH, USA; 0990), Neomycin (Sigma-Aldrich, MO, USA; N6386), Metronidazole (Sigma-Aldrich, MO, USA;M1547).

### Animals, treatments and sampling

C57BL6/J male mice (n = 122) were purchased from Jackson Laboratory at the age of 8–10 weeks and group housed (4/5-mice per cage) in the centralized animal care facility of the Center for Comparative Medicine and Surgery at the Icahn School of Medicine at Mount Sinai. All mice were maintained on a 12:12-h light/dark cycle with lights on at 07:00 h in a temperature-controlled (20 ± 2 °C) vivarium. Mice were allowed to adapt to the new environment for at least 2 weeks and were tested at 3–4 months of age. Mice were fed a polyphenol free diet. For assessing the gut microbiota’s role in dietary polyphenols’ effect in a model of SD-induced memory impairment, following 2-weeks of polyphenol-free diet, mice were randomly subdivided for six treatment groups including: non sleep-deprived mice receiving regular drinking water (n = 20, Ctrl), sleep-deprived mice treated with regular drinking water (n = 20, SD), sleep-deprived mice treated with BDPP for 13 days (n = 21, SD + BDPP), non sleep-deprived mice treated with ABX mix for 23 days (n = 19, ABX), sleep-deprived mice treated with ABX mix for 23 days (n = 20, SD + ABX), and sleep-deprived mice treated with ABX for 10 days, and then BDPP supplemented with ABX for another 13 days (n = 22, SD + ABX + BDPP). ABX mix, composed of Ampicillin (1 mg/ml), Vancomycin (0.5 mg/ml), Neomycin (1 mg/ml) and Metronidazole (1 mg/ml) with the addition of Splenda (artificial sweetener, 1 mg/ml) to improve palatability of water, was delivered through the drinking water for 23 days prior and throughout behavioral testing. BDPP, composed of GSPE, RESV and CGJ, was delivered through the drinking water for 13 days prior to and throughout behavioral testing. The calculated daily intake of GSE was 200 mg/kg body weight (BW)^1,16^, resveratrol was 400 mg/kg BW^44,45^ and the total polyphenols from juice extract was 183 mg/kg BW^46^. These doses were chosen based on the equivalent doses used in a previous study that showed efficacy in animal models for each component^17^. ABX mix was changed once a week. BDPP was changed once every two days. To study treatment effect on memory consolidation, n = 7–10 mice per group were sacrificed by CO_2_ euthanasia at the end of the 5 h sleep deprivation that followed behavioral training (Day 22) and blood samples were collected through cardiac puncture by inserting a 23 G needle into the ventricle. Plasma was recovered following centrifugation, acidified with formic acid (1:10 vol/vol, with the final concentration of formic acid 0.2%) and stored at −80 °C. To investigate treatment effect on gut bacterial population, feces were collected on day 21. Since fecal collection took place before the SD procedure, gut microbiota analyses was conducted in the following treatment groups: Ctrl; mice from Ctrl and SD groups, BDPP; mice from SD + BDPP group, ABX; mice from ABX and SD + ABX groups; ABX + BDPP; mice from SD + ABX + BDPP group. For all experiments, mice body weight and food consumption was assessed once a week. Liquid consumption was assessed every 2 days. Data is summarized in Supplemental Fig. 1. All procedures were approved by the Mount Sinai IACUC and conducted in accordance with the guidelines and regulations.

### Sleep deprivation

Mice were sleep deprived by using the Automated Sleep Deprivation System for Mice, Pinnacle technologies Inc., KS, USA^47,48^. The system uses timed movements of the randomly rotating bar to prevent mice from sleeping. Mice were acclimated to the Automated Sleep Deprivation System for 30 min/day for 3 consecutive days prior to the sleep deprivation procedure. For memory consolidation examination, mice were maintained in the Automated Sleep Deprivation System for 5 h starting immediately after a 10 min training trial. For each system, n = 5 mice from the same home cage were housed together with ad lib access to food and treatments containing drinking bottles. At the end of the 5 h SD mice were either sacrificed for tissue collection or placed back in the home cage for the next day behavioral testing.

### Cognitive assessment of SD-induced memory impairment

For characterizations of the gut microbiota’s role in polyphenols’ treatment effect on SD-induced memory impairment, we conducted a contextual fear conditioning test as previously described^49^. On Day 1, mice were placed into a fear conditioning chamber (Med Associates, 30.5 × 24.1 × 21 cm) containing gray walls, grid floor, houselights at 50% with lemon scent, and allowed to explore for 120 s prior to 2 s foot shock (0.7 mA) from the metal grid and then for additional 30 s. After the training session on Day 1, non-sleep deprived mice were left undisturbed in their home cages, and sleep-deprived mice were kept awake by the Automated Sleep Deprivation System. On Day 2, 24-h after the training session, mice were placed into the same context and allowed to explore for 120 s without the shock. Memory for the context (contextual memory) for each animal was obtained by measuring freezing behavior, defined by body immobility except for respiration. Freezing behavior was scored over the 2 min testing period and data were presented as a percentage of time in freezing over total sampling period. Freezing behavior was recorded with a side-mounted NIR camera and measured with ANY-maze™ tracking software (Stoelting Co., IL, USA. Version 5.1 Beta). All animals were handled for 5 min/day for 5 days prior to behavioral testing. Mice were habituated to the testing room for 30 min at the beginning of training and test day. All tests were conducted by the same experimenter.

### Phenolic acid metabolites extraction from plasma samples

Fifty-one plasma samples were collected on day 22 and divided into treatment groups: Ctrl (n = 8); SD (n = 8); SD + BDPP (n = 5); ABX (n = 10); SD + ABX (n = 10); SD + ABX + BDPP (n = 10), were stored at −80 °C prior to analysis. Plasma samples were extracted based on previously published methods^50,51^ with minor modification. Briefly, plasma (140 μL) samples were incubated, 1 mL enzyme solution (250 U *β*-glucuronidase + sulfatase contamination in 0.4 M NaH_2_PO_4_pH 4.5) for 45 min at 37 °C after purging with nitrogen. After incubation, all samples were acidified to pH < 3 by hydrochloric acid, followed by extraction three times with ethyl acetate/0.01% 2, 6-di-tert-butyl-4-methylphenol. Combined ethyl acetate fractions were dried under vacuum, re-solubilized in 200 μL mobile phase (90% mobile phase A and 10% mobile phase B), and filtrated through a 0.45 μm Nylon SYR filter (National Scientific, Rockwood, TN) prior to analysis.

### 16S rRNA gene amplicon sequencing

Stool samples of mice in treatment groups (Ctrl, BDPP, ABX, ABX + BDPP) were collected under the same condition on Day 21 (prior to SD or behavioral testing). Each mouse fecal pellet was weighed. Fecal DNA was extracted by bead-beating and quantified with Qubit assays. The 16S rDNA amplicon sequencing of the V4 hypervariable region was performed with an Illumina MiSeq (paired-end 250 bp). For each PCR reaction, 4 ng of purified template DNA was amplified in a reaction volume of 20 μl. Primers were designed to target the V4 region of the 16S rDNA (position 515 of the bacterial 16S rRNA gene to position 806). Each reaction was denatured at 98 °C for 30 sec followed by 26 cycles of (98 °C × 10 sec, 50 °C × 30 sec, 72 °C × 30 sec), followed by a final extension at 72 °C for 2 min. Reactions each contained a unique sequence index to enable pooling. Pools were purified with 60 μl AmpureXP beads added to 100 μl of sample (i.e., a beads to sample ratio of 0.6) and sequenced on an Illumina MiSeq instrument at a loading concentration of 10 pM. The 16S rDNA data were analyzed with QIIME 1.9.1 for taxonomic classification analysis of OTUs^52^.

A training dataset for taxonomic assignments was created using a modified NCBI taxonomy from the ‘isolated named strains 16S’ in the Greengenes database^53^. This dataset was manually curated by (i) removing strains in ‘isolated named strains 16S’ that had non-standard taxonomy or that were not members of the domain bacteria, and (ii) grouping strain level taxonomy from Greengenes assignments under a single NCBI species assignment. The trained dataset is available at http://gordonlab.wustl.edu/SuppData.html and was used to train the Ribosomal Database Project (RDP) version 2.4 (56) classifier and to assign taxonomy to picked OTUs.

Shannon indices, indicative of α-diversity, were then calculated from refaction plots. Weighted UniFrac distances were then computed to produce β-diversity dissimilarity matrices^54^.

### Statistical analyses

For statistical analysis of fear conditioning freezing behavior and Shannon index for gut microbial α-diversity comparing treatments groups, one-way ANOVA followed by Tukey multiple comparisons was performed. Differences in phenolic acid plasma concentration collected on Day 22 in SD + BDPP vs. ABX + SD + BDPP group were analyzed by Wilcoxon rank test. Significantly different was defined as Benjamin-Hochberg false discovery rate (FDR) < 10.7%. Principal component analyses (PCA) was conducted for analyses of differences in the composition of OTUs among treatment groups. Each mouse data was projected to the space of the first 2 principle components. Association of OTUs composition with freezing behavior or with phenolic acids bioavailability was conducted with Univariate linear regression. For OTUs association with phenolic acids bioavailability, a quantile-quantile (QQ) plot was generated to visualize the distribution of the p-values, comparing the observed p-values against expected p-values under the null hypothesis (ie, no association). All values are expressed as mean and standard error of the mean (s.e.m). Statistical analysis was performed using GraphPad Prism 5 software (GraphPad Software, Inc.) and R statistical software R 3.0.2.

## Supplementary information

Supplementary information

## Data Availability

The datasets generated during and/or analyzed during the current study are available from the corresponding author on reasonable request.
