# Supplementary material for: The gut microbiota composition affects dietary polyphenols-mediated cognitive resilience in mice by modulating the bioavailability of phenolic acids
Source: Sci Rep. 2019 Mar 5;9:3546. doi: 10.1038/s41598-019-39994-6 (PMC6401062; doi:10.1038/s41598-019-39994-6)
Supplement: Supplementary file 1 — Supplementary information [file 41598_2019_39994_MOESM1_ESM.docx]

**SUPPLEMENTARY INFORMATION**

**Title:**

The gut microbiota composition affects dietary polyphenols-mediated cognitive resilience in mice by modulating the bioavailability of phenolic acids.

**Authors:**

Tal Frolinger^1^, Steven Sims^1^, Chad Smith^1^, Jun Wang^1^, Haoxiang Cheng^3, 4^, Jeremiah Faith^3^, Lap Ho^1^, Ke Hao^3,4^, *Giulio M. Pasinetti^1, 2^

**Supplementary Fig. S1**

**
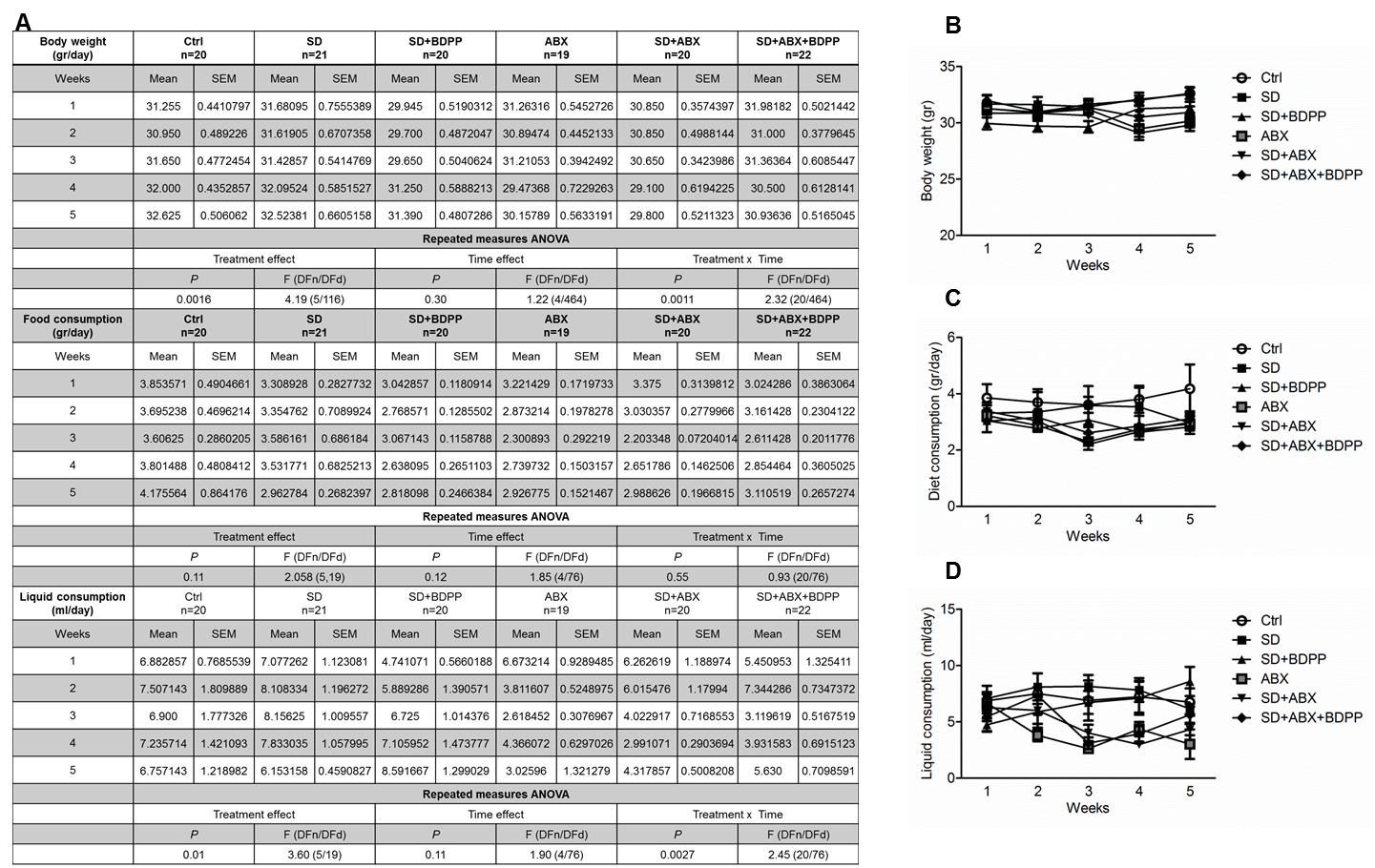
**

**Supplementary Fig S1.**Physiological data. 1B) Body weight (gr/week), diet consumption (gr/day) and liquid consumption (ml/day) in non-sleep deprived-vehicle treated mice (Ctrl), sleep deprived-vehicle treated mice (SD), sleep deprived-BDPP treated mice (SD+BDPP), non-sleep deprived-antibiotics treated mice (ABX), sleep deprived-antibiotics treated mice (SD) and sleep deprived-antibiotics and BDPP treated mice (SD+ABX+BDPP). Values are mean ± SEM. Repeated measures ANOVA was used to calculate *p* values. B, C, D) Graphic presentation of Body weight gain (gr/week), diet consumption (gr/day) and liquid consumption (ml/day) across the 4-weeks treatment regimen. Data is mean ± SEM (Repeated measures ANOVA).

**Supplementary Fig. S2**

**
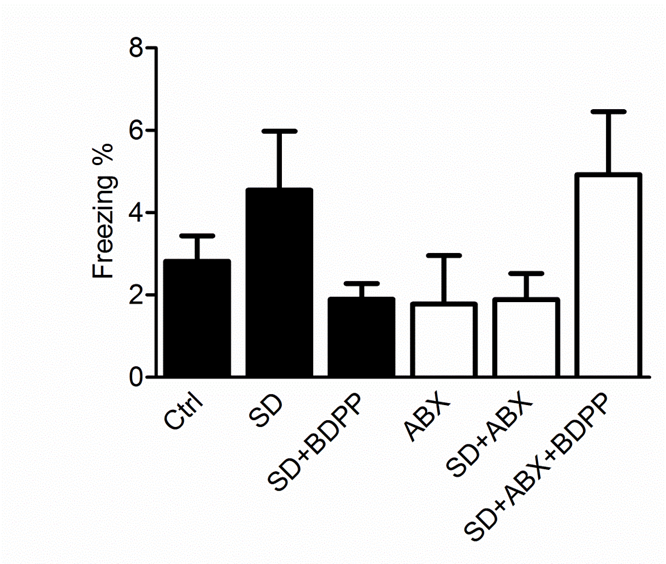
**

**Supplementary Fig S2. Basal freezing behavior.** Neither ABX nor BDPP treatments affect freezing behavior in training trial. C57BL6 mice were randomly grouped into 6 groups: non sleep-deprived vehicle treated (Ctrl), sleep-deprived vehicle treated (SD), sleep-deprived BDPP treated (SD+BDPP), non sleep-deprived antibiotics treated (ABX), sleep-deprived antibiotics treated (SD+ABX), sleep-deprived treated with antibiotics in addition to BDPP (SD+ABX+BDPP). Basal freezing percent across 120s of contextual fear conditioning training trial prior to foot shock was recorded. Data are means + SEM (1-Way ANOVA; 5,62, F=1,68, p=0.163, Tukey's Multiple Comparison Test, p>0.05).

**Supplementary Table S1**.

**Supplementary Table S1**. **Phenolic acid concentration.** Mean ± standard deviation of the plasma phenolic acid concentration (ng/ml) collected on Day 22 in non sleep-deprived vehicle treated (Ctrl), sleep-deprived vehicle treated (SD), sleep-deprived BDPP treated (SD+BDPP), non sleep-deprived antibiotics treated (ABX), sleep-deprived antibiotics treated (SD+ABX), sleep-deprived treated with antibiotics in addition to BDPP (SD+ABX+BDPP). Wilcoxon Test p-value and false discovery rate (FDR) comparison of Day 22 phenolic acids plasma concentration in SD+BDPP vs, SD+ABX+BDPP groups were calculated. Significant differences (p<0.01 at FDR≤0.107) are highlighted in red.

**Supplementary Table S2**.

**Supplementary Table S2**. **Taxa summary.** A list of the 34 genus of OTUs identified in fecal contents, collected at Day 21, from male C57BL6 mice in non sleep-deprived vehicle treated (Ctrl, *n*=15), non sleep-deprived BDPP treated (BDPP, *n*=9), non sleep-deprived antibiotics treated (ABX, *n*=33) and non sleep-deprived treated with antibiotics in addition to BDPP (ABX+BDPP, *n*=17) treatment groups and their relative percentage of abundance in total samples and in each of the treatment group. OTUs are classified by: Kingdom (K), Phylum (P), Class (C), Order (O), Family (F) and Genus (G).

**Supplementary Table S3**.

**Supplementary Table S3**. **Taxonomic classification analyses.** Mean ± standard deviation of the unnormalized counts of 62 OTUs identified in fecal contents, collected at Day 21, from male C57BL6 mice in non sleep-deprived vehicle treated (Ctrl, *n*=15), non sleep-deprived BDPP treated (BDPP, *n*=9), non sleep-deprived antibiotics treated (ABX, *n*=33) and non sleep-deprived treated with antibiotics in addition to BDPP (ABX+BDPP, *n*=17) treatment groups. OTUs are classified by: Kingdom (K), Phylum (P), Class (C), Order (O), Family (F) and Genus (G). Wilcoxon Test p-value and false discovery rate (FDR) comparison of OTUs counts in ABX vs Ctrl, ABX+BDPP vs. BDPP and BDPP vs. Ctrl groups were calculated. Significant differences (p<0.01 at FDR≤0.107) are highlighted in red.

**Supplementary Table S4.**

**Supplementary Table S4. Shannon index.** [QIIME](http://www.wernerlab.org/teaching/qiime)‎ α-diversity calculation (average (Ave) and Standard error (Err) ; by the PD_ whole _tree, observed_ species and Shannon matrices for each number of sequences per sample. Fecal contents were collected at Day 21, from male C57BL6 mice in non sleep-deprived vehicle treated (Ctrl), non sleep-deprived BDPP treated (BDPP), non sleep-deprived antibiotics treated (ABX) and non sleep-deprived treated with antibiotics in addition to BDPP (ABX+BDPP) treatment groups.

**Supplementary Table S5.**

**Supplementary Table S5. Association of OTUs composition with freezing behavior.** OTUs’ differences’ proportion of association (p-values) to freezing behavior differences in fear conditioning memory test was analyzed by Univariate linear regression in ABX vs Ctrl, SD+BDPP vs. SD and ABX+SD+BDPP vs. SD+BDPP. Significant differences (p<0.01 at FDR≤0.107) are highlighted in red. (-), negative association.

**Supplementary Table S6.**

| Pair-wise correlations of plasma phenolic acid's concentration diference - OTU abandance diference ABX SD+BDPP and SD+BDPP groups |  |  |  |  |  |  |
| --- | --- | --- | --- | --- | --- | --- |
| **OTUs Taxonomy [Kingdom (k) , Phylum (p), Class (c), Order (o), Family (f), Genus (g) and Species (s)]** | Phenolic Acid | estimate | std.error | statistic | p.value | FDR |
| k__Bacteria; p__Firmicutes; c__Bacilli; o__Lactobacillales; f__Aerococcaceae; g__; s__ | HVA | -0.159 | 0.050 | -3.156 | 0.002 | 0.015 |
| k__Bacteria; p__Firmicutes; c__Bacilli; o__Bacillales; f__Planococcaceae; g__Sporosarcina; s__ | HVA | -0.164 | 0.058 | -2.852 | 0.006 | 0.027 |
| k__Bacteria; p__Firmicutes; c__Bacilli; o__Bacillales; f__Planococcaceae; g__Sporosarcina; s__ | HVA | -0.121 | 0.044 | -2.721 | 0.008 | 0.035 |
| k__Bacteria; p__Firmicutes; c__Bacilli; o__Bacillales; f__Planococcaceae; g__Sporosarcina; s__ | HVA | -0.141 | 0.052 | -2.706 | 0.009 | 0.036 |
| k__Bacteria; p__Proteobacteria; c__Gammaproteobacteria; o__Alteromonadales; f__Shewanellaceae; g__Shewanella; s__algae | HVA | -0.129 | 0.056 | -2.293 | 0.025 | 0.075 |
| k__Bacteria; p__Proteobacteria; c__Gammaproteobacteria; o__Xanthomonadales; f__Xanthomonadaceae; g__Stenotrophomonas; s__ | HVA | -0.057 | 0.027 | -2.078 | 0.042 | 0.110 |
| k__Bacteria; p__Proteobacteria; c__Betaproteobacteria; o__Burkholderiales; f__Alcaligenaceae; g__Achromobacter; s__ | HVA | -0.104 | 0.051 | -2.057 | 0.044 | 0.114 |
| k__Bacteria; p__Proteobacteria; c__Gammaproteobacteria; o__Pseudomonadales; f__Pseudomonadaceae; g__Pseudomonas; s__ | HVA | -0.153 | 0.076 | -2.008 | 0.049 | 0.119 |
| k__Bacteria; p__Proteobacteria; c__Gammaproteobacteria; o__Xanthomonadales; f__Sinobacteraceae; g__Nevskia; s__ | HVA | -0.184 | 0.094 | -1.956 | 0.055 | 0.130 |
| k__Bacteria; p__Proteobacteria; c__Gammaproteobacteria; o__Pseudomonadales; f__Pseudomonadaceae; g__Pseudomonas; s__veronii | HVA | -0.057 | 0.030 | -1.929 | 0.058 | 0.134 |
| k__Bacteria; p__Proteobacteria; c__Gammaproteobacteria; o__Pseudomonadales; f__Pseudomonadaceae; g__Pseudomonas; s__veronii | HVA | -6.725 | 3.507 | -1.918 | 0.060 | 0.136 |
| k__Bacteria; p__Actinobacteria; c__Coriobacteriia; o__Coriobacteriales; f__Coriobacteriaceae; g__; s__ | HVA | 4.093 | 2.148 | 1.906 | 0.061 | 0.136 |
| k__Bacteria; p__Proteobacteria; c__Gammaproteobacteria; o__Pseudomonadales; f__Pseudomonadaceae; g__Pseudomonas; s__ | HVA | -0.069 | 0.040 | -1.730 | 0.088 | 0.183 |
| k__Bacteria; p__Proteobacteria; c__Gammaproteobacteria; o__Oceanospirillales; f__Halomonadaceae; g__Halomonas; s__ | HVA | -0.121 | 0.072 | -1.688 | 0.096 | 0.193 |
| k__Bacteria; p__Firmicutes; c__Erysipelotrichi; o__Erysipelotrichales; f__Erysipelotrichaceae; g__; s__ | HVA | 0.865 | 0.522 | 1.657 | 0.102 | 0.201 |
| k__Bacteria; p__Bacteroidetes; c__[Saprospirae]; o__[Saprospirales]; f__Chitinophagaceae; g__Sediminibacterium; s__ | HVA | -0.026 | 0.016 | -1.608 | 0.113 | 0.215 |
| k__Bacteria; p__Proteobacteria; c__Alphaproteobacteria; o__Caulobacterales; f__Caulobacteraceae; g__; s__ | HVA | -0.052 | 0.034 | -1.521 | 0.133 | 0.240 |
| k__Bacteria; p__Actinobacteria; c__Coriobacteriia; o__Coriobacteriales; f__Coriobacteriaceae; g__Adlercreutzia; s__ | HVA | 0.190 | 0.126 | 1.504 | 0.137 | 0.246 |
| k__Bacteria; p__Proteobacteria; c__Alphaproteobacteria; o__Rhizobiales; f__Bradyrhizobiaceae; g__Bradyrhizobium; s__ | HVA | -0.032 | 0.024 | -1.307 | 0.196 | 0.317 |
| k__Bacteria; p__Firmicutes; c__Clostridia; o__Clostridiales; f__Lachnospiraceae; g__; s__ | HVA | 0.547 | 0.421 | 1.299 | 0.199 | 0.318 |
| k__Bacteria; p__Proteobacteria; c__Gammaproteobacteria; o__Oceanospirillales; f__Halomonadaceae; g__Halomonas; s__ | HVA | -0.068 | 0.053 | -1.291 | 0.201 | 0.318 |
| k__Bacteria; p__Firmicutes; c__Clostridia; o__Clostridiales; f__; g__; s__ | HVA | 0.072 | 0.056 | 1.284 | 0.204 | 0.320 |
| k__Bacteria; p__Firmicutes; c__Clostridia; o__Clostridiales; f__Ruminococcaceae; g__; s__ | HVA | 0.069 | 0.055 | 1.259 | 0.213 | 0.327 |
| k__Bacteria; p__Firmicutes; c__Clostridia; o__Clostridiales; f__; g__; s__ | HVA | 0.483 | 0.387 | 1.248 | 0.217 | 0.331 |
| k__Bacteria; p__Firmicutes; c__Bacilli; o__Lactobacillales; f__Streptococcaceae; g__Streptococcus; s__ | HVA | -0.284 | 0.230 | -1.235 | 0.221 | 0.334 |
| k__Bacteria; p__Firmicutes; c__Clostridia; o__Clostridiales; f__; g__; s__ | HVA | 0.174 | 0.142 | 1.220 | 0.227 | 0.334 |
| k__Bacteria; p__Verrucomicrobia; c__Verrucomicrobiae; o__Verrucomicrobiales; f__Verrucomicrobiaceae; g__Akkermansia; s__muciniphila | HVA | 1.642 | 1.347 | 1.219 | 0.227 | 0.334 |
| k__Bacteria; p__Actinobacteria; c__Coriobacteriia; o__Coriobacteriales; f__Coriobacteriaceae; g__Adlercreutzia; s__ | HVA | 0.105 | 0.087 | 1.219 | 0.227 | 0.334 |
| k__Bacteria; p__Firmicutes; c__Bacilli; o__Lactobacillales; f__Aerococcaceae; g__Aerococcus; s__ | HVA | 0.302 | 0.267 | 1.130 | 0.263 | 0.366 |
| k__Bacteria; p__Firmicutes; c__Clostridia; o__Clostridiales; f__Lachnospiraceae; g__; s__ | HVA | 0.198 | 0.176 | 1.128 | 0.264 | 0.366 |
| k__Bacteria; p__Firmicutes; c__Clostridia; o__Clostridiales; f__Ruminococcaceae; g__; s__ | HVA | 0.199 | 0.203 | 0.982 | 0.330 | 0.402 |
| k__Bacteria; p__Firmicutes; c__Clostridia; o__Clostridiales; f__; g__; s__ | HVA | 0.072 | 0.075 | 0.956 | 0.343 | 0.402 |
| k__Bacteria; p__Firmicutes; c__Clostridia; o__Clostridiales; f__Ruminococcaceae; g__; s__ | HVA | 0.058 | 0.065 | 0.888 | 0.378 | 0.402 |
| k__Bacteria; p__Firmicutes; c__Clostridia; o__Clostridiales; f__Lachnospiraceae; g__Dorea; s__ | HVA | 0.088 | 0.099 | 0.888 | 0.378 | 0.402 |
| k__Bacteria; p__Firmicutes; c__Clostridia; o__Clostridiales; f__Lachnospiraceae; g__; s__ | HVA | 0.230 | 0.266 | 0.863 | 0.391 | 0.404 |
| k__Bacteria; p__Bacteroidetes; c__Bacteroidia; o__Bacteroidales; f__S24-7; g__; s__ | HVA | 1.489 | 2.286 | 0.652 | 0.517 | 0.445 |
| k__Bacteria; p__Bacteroidetes; c__Bacteroidia; o__Bacteroidales; f__S24-7; g__; s__ | HVA | 0.037 | 0.060 | 0.606 | 0.547 | 0.446 |
| k__Bacteria; p__Bacteroidetes; c__Bacteroidia; o__Bacteroidales; f__S24-7; g__; s__ | HVA | 0.047 | 0.077 | 0.604 | 0.548 | 0.446 |
| k__Bacteria; p__Bacteroidetes; c__Bacteroidia; o__Bacteroidales; f__S24-7; g__; s__ | HVA | 0.941 | 1.567 | 0.601 | 0.550 | 0.446 |
| k__Bacteria; p__Firmicutes; c__Bacilli; o__Lactobacillales; f__Enterococcaceae; g__; s__ | HVA | -0.054 | 0.092 | -0.586 | 0.560 | 0.446 |
| k__Bacteria; p__Firmicutes; c__Clostridia; o__Clostridiales; f__Clostridiaceae; g__; s__ | HVA | 0.019 | 0.044 | 0.430 | 0.669 | 0.471 |
| k__Bacteria; p__Proteobacteria; c__Gammaproteobacteria; o__Enterobacteriales; f__Enterobacteriaceae; g__; s__ | HVA | 0.657 | 1.588 | 0.414 | 0.680 | 0.471 |
| k__Bacteria; p__Actinobacteria; c__Actinobacteria; o__Bifidobacteriales; f__Bifidobacteriaceae; g__Bifidobacterium; s__ | HVA | 0.029 | 0.073 | 0.401 | 0.690 | 0.471 |
| k__Bacteria; p__Actinobacteria; c__Actinobacteria; o__Bifidobacteriales; f__Bifidobacteriaceae; g__Bifidobacterium; s__pseudolongum | HVA | 2.010 | 5.040 | 0.399 | 0.691 | 0.471 |
| k__Bacteria; p__Firmicutes; c__Bacilli; o__Lactobacillales; f__Enterococcaceae; g__Enterococcus; s__ | HVA | 0.240 | 0.632 | 0.379 | 0.706 | 0.474 |
| k__Bacteria; p__Firmicutes; c__Bacilli; o__Turicibacterales; f__Turicibacteraceae; g__Turicibacter; s__ | HVA | 0.045 | 0.130 | 0.347 | 0.729 | 0.478 |
| k__Bacteria; p__Firmicutes; c__Bacilli; o__Bacillales; f__Staphylococcaceae; g__Staphylococcus; s__succinus | HVA | 0.046 | 0.164 | 0.280 | 0.780 | 0.491 |
| k__Bacteria; p__Actinobacteria; c__Coriobacteriia; o__Coriobacteriales; f__Coriobacteriaceae; g__Adlercreutzia; s__ | HVA | -0.028 | 0.106 | -0.266 | 0.791 | 0.496 |
| k__Bacteria; p__Firmicutes; c__Bacilli; o__Lactobacillales; f__Lactobacillaceae; g__Lactobacillus; s__ | HVA | 0.439 | 1.747 | 0.251 | 0.802 | 0.499 |
| k__Bacteria; p__Firmicutes; c__Bacilli; o__Lactobacillales; f__Lactobacillaceae; g__Lactobacillus; s__ | HVA | 0.021 | 0.100 | 0.208 | 0.836 | 0.509 |
| k__Bacteria; p__Firmicutes; c__Clostridia; o__Clostridiales; f__; g__; s__ | HVA | -0.022 | 0.129 | -0.168 | 0.867 | 0.517 |
| k__Bacteria; p__Proteobacteria; c__Gammaproteobacteria; o__Enterobacteriales; f__Enterobacteriaceae; g__; s__ | HVA | 0.020 | 0.126 | 0.158 | 0.875 | 0.518 |
| k__Bacteria; p__Proteobacteria; c__Gammaproteobacteria; o__Enterobacteriales; f__Enterobacteriaceae; g__Pantoea; s__agglomerans | HVA | 0.581 | 3.732 | 0.156 | 0.877 | 0.518 |
| k__Bacteria; p__Firmicutes; c__Erysipelotrichi; o__Erysipelotrichales; f__Erysipelotrichaceae; g__Allobaculum; s__ | HVA | 0.023 | 0.163 | 0.143 | 0.886 | 0.518 |
| k__Bacteria; p__Firmicutes; c__Bacilli; o__Lactobacillales; f__Lactobacillaceae; g__Lactobacillus; s__ | HVA | -0.196 | 1.503 | -0.130 | 0.897 | 0.518 |
| k__Bacteria; p__Firmicutes; c__Bacilli; o__Bacillales; f__Staphylococcaceae; g__Staphylococcus; s__sciuri | HVA | -0.090 | 0.775 | -0.116 | 0.908 | 0.522 |
| k__Bacteria; p__Firmicutes; c__Bacilli; o__Lactobacillales; f__Lactobacillaceae; g__Lactobacillus; s__ | HVA | -0.006 | 0.060 | -0.096 | 0.924 | 0.526 |
| k__Bacteria; p__Proteobacteria; c__Gammaproteobacteria; o__Enterobacteriales; f__Enterobacteriaceae; g__; s__ | HVA | 0.011 | 0.131 | 0.082 | 0.935 | 0.529 |
| k__Bacteria; p__Firmicutes; c__Clostridia; o__Clostridiales; f__; g__; s__ | HVA | 0.006 | 0.107 | 0.055 | 0.956 | 0.534 |
| k__Bacteria; p__Bacteroidetes; c__Bacteroidia; o__Bacteroidales; f__S24-7; g__; s__ | HVA | -0.005 | 0.135 | -0.040 | 0.968 | 0.538 |
| k__Bacteria; p__Firmicutes; c__Clostridia; o__Clostridiales; f__Peptostreptococcaceae; g__; s__ | HVA | 0.004 | 0.143 | 0.026 | 0.979 | 0.540 |
| k__Bacteria; p__Firmicutes; c__Bacilli; o__Bacillales; f__Staphylococcaceae; g__Jeotgalicoccus; s__psychrophilus | HVA | 0.006 | 0.287 | 0.022 | 0.982 | 0.540 |
| k__Bacteria; p__Proteobacteria; c__Gammaproteobacteria; o__Pseudomonadales; f__Pseudomonadaceae; g__Pseudomonas; s__veronii | HA | -13.350 | 1.661 | -8.040 | 0.000 | 0.000 |
| k__Bacteria; p__Proteobacteria; c__Gammaproteobacteria; o__Xanthomonadales; f__Sinobacteraceae; g__Nevskia; s__ | HA | -0.352 | 0.044 | -7.949 | 0.000 | 0.000 |
| k__Bacteria; p__Proteobacteria; c__Gammaproteobacteria; o__Xanthomonadales; f__Xanthomonadaceae; g__Stenotrophomonas; s__ | HA | -0.097 | 0.012 | -7.893 | 0.000 | 0.000 |
| k__Bacteria; p__Proteobacteria; c__Gammaproteobacteria; o__Oceanospirillales; f__Halomonadaceae; g__Halomonas; s__ | HA | -0.261 | 0.035 | -7.473 | 0.000 | 0.000 |
| k__Bacteria; p__Proteobacteria; c__Betaproteobacteria; o__Burkholderiales; f__Alcaligenaceae; g__Achromobacter; s__ | HA | -0.184 | 0.025 | -7.424 | 0.000 | 0.000 |
| k__Bacteria; p__Proteobacteria; c__Gammaproteobacteria; o__Pseudomonadales; f__Pseudomonadaceae; g__Pseudomonas; s__ | HA | -0.145 | 0.020 | -7.232 | 0.000 | 0.000 |
| k__Bacteria; p__Proteobacteria; c__Gammaproteobacteria; o__Alteromonadales; f__Shewanellaceae; g__Shewanella; s__algae | HA | -0.201 | 0.029 | -6.856 | 0.000 | 0.000 |
| k__Bacteria; p__Proteobacteria; c__Gammaproteobacteria; o__Pseudomonadales; f__Pseudomonadaceae; g__Pseudomonas; s__ | HA | -0.267 | 0.039 | -6.851 | 0.000 | 0.000 |
| k__Bacteria; p__Proteobacteria; c__Gammaproteobacteria; o__Oceanospirillales; f__Halomonadaceae; g__Halomonas; s__ | HA | -0.178 | 0.027 | -6.704 | 0.000 | 0.000 |
| k__Bacteria; p__Proteobacteria; c__Alphaproteobacteria; o__Caulobacterales; f__Caulobacteraceae; g__; s__ | HA | -0.118 | 0.018 | -6.658 | 0.000 | 0.000 |
| k__Bacteria; p__Bacteroidetes; c__[Saprospirae]; o__[Saprospirales]; f__Chitinophagaceae; g__Sediminibacterium; s__ | HA | -0.057 | 0.009 | -6.643 | 0.000 | 0.000 |
| k__Bacteria; p__Proteobacteria; c__Alphaproteobacteria; o__Rhizobiales; f__Bradyrhizobiaceae; g__Bradyrhizobium; s__ | HA | -0.082 | 0.013 | -6.574 | 0.000 | 0.000 |
| k__Bacteria; p__Bacteroidetes; c__Bacteroidia; o__Bacteroidales; f__S24-7; g__; s__ | HA | 7.221 | 1.100 | 6.565 | 0.000 | 0.000 |
| k__Bacteria; p__Firmicutes; c__Clostridia; o__Clostridiales; f__Ruminococcaceae; g__; s__ | HA | 0.641 | 0.100 | 6.385 | 0.000 | 0.000 |
| k__Bacteria; p__Firmicutes; c__Clostridia; o__Clostridiales; f__Clostridiaceae; g__; s__ | HA | 0.137 | 0.023 | 6.051 | 0.000 | 0.000 |
| k__Bacteria; p__Actinobacteria; c__Coriobacteriia; o__Coriobacteriales; f__Coriobacteriaceae; g__; s__ | HA | 6.877 | 1.139 | 6.037 | 0.000 | 0.000 |
| k__Bacteria; p__Firmicutes; c__Erysipelotrichi; o__Erysipelotrichales; f__Erysipelotrichaceae; g__; s__ | HA | 0.427 | 0.085 | 5.037 | 0.000 | 0.000 |
| k__Bacteria; p__Bacteroidetes; c__Bacteroidia; o__Bacteroidales; f__S24-7; g__; s__ | HA | 2.379 | 0.545 | 4.369 | 0.000 | 0.000 |
| k__Bacteria; p__Firmicutes; c__Clostridia; o__Clostridiales; f__Ruminococcaceae; g__; s__ | HA | 0.131 | 0.032 | 4.051 | 0.000 | 0.001 |
| k__Bacteria; p__Actinobacteria; c__Coriobacteriia; o__Coriobacteriales; f__Coriobacteriaceae; g__Adlercreutzia; s__ | HA | 0.159 | 0.040 | 3.955 | 0.000 | 0.002 |
| k__Bacteria; p__Firmicutes; c__Clostridia; o__Clostridiales; f__; g__; s__ | HA | 0.306 | 0.079 | 3.875 | 0.000 | 0.002 |
| k__Bacteria; p__Bacteroidetes; c__Bacteroidia; o__Bacteroidales; f__S24-7; g__; s__ | HA | 0.120 | 0.032 | 3.780 | 0.000 | 0.003 |
| k__Bacteria; p__Verrucomicrobia; c__Verrucomicrobiae; o__Verrucomicrobiales; f__Verrucomicrobiaceae; g__Akkermansia; s__muciniphila | HA | 1.749 | 0.473 | 3.700 | 0.000 | 0.004 |
| k__Bacteria; p__Firmicutes; c__Clostridia; o__Clostridiales; f__Lachnospiraceae; g__; s__ | HA | 0.073 | 0.021 | 3.529 | 0.001 | 0.006 |
| k__Bacteria; p__Firmicutes; c__Clostridia; o__Clostridiales; f__; g__; s__ | HA | 0.077 | 0.022 | 3.483 | 0.001 | 0.006 |
| k__Bacteria; p__Firmicutes; c__Bacilli; o__Lactobacillales; f__Aerococcaceae; g__Aerococcus; s__ | HA | 0.555 | 0.161 | 3.454 | 0.001 | 0.007 |
| k__Bacteria; p__Actinobacteria; c__Actinobacteria; o__Bifidobacteriales; f__Bifidobacteriaceae; g__Bifidobacterium; s__pseudolongum | HA | 10.428 | 3.042 | 3.428 | 0.001 | 0.007 |
| k__Bacteria; p__Actinobacteria; c__Actinobacteria; o__Bifidobacteriales; f__Bifidobacteriaceae; g__Bifidobacterium; s__ | HA | 0.150 | 0.044 | 3.419 | 0.001 | 0.007 |
| k__Bacteria; p__Firmicutes; c__Clostridia; o__Clostridiales; f__Lachnospiraceae; g__Dorea; s__ | HA | 0.190 | 0.057 | 3.333 | 0.001 | 0.009 |
| k__Bacteria; p__Actinobacteria; c__Coriobacteriia; o__Coriobacteriales; f__Coriobacteriaceae; g__Adlercreutzia; s__ | HA | 0.145 | 0.044 | 3.266 | 0.002 | 0.011 |
| k__Bacteria; p__Firmicutes; c__Bacilli; o__Lactobacillales; f__Lactobacillaceae; g__Lactobacillus; s__ | HA | 3.237 | 1.062 | 3.049 | 0.003 | 0.019 |
| k__Bacteria; p__Firmicutes; c__Clostridia; o__Clostridiales; f__Lachnospiraceae; g__; s__ | HA | 0.205 | 0.069 | 2.968 | 0.004 | 0.023 |
| k__Bacteria; p__Firmicutes; c__Clostridia; o__Clostridiales; f__; g__; s__ | HA | 0.231 | 0.078 | 2.947 | 0.004 | 0.023 |
| k__Bacteria; p__Firmicutes; c__Bacilli; o__Lactobacillales; f__Lactobacillaceae; g__Lactobacillus; s__ | HA | 0.171 | 0.062 | 2.780 | 0.007 | 0.032 |
| k__Bacteria; p__Firmicutes; c__Bacilli; o__Bacillales; f__Staphylococcaceae; g__Staphylococcus; s__succinus | HA | 0.270 | 0.101 | 2.683 | 0.009 | 0.038 |
| k__Bacteria; p__Firmicutes; c__Clostridia; o__Clostridiales; f__; g__; s__ | HA | 0.087 | 0.033 | 2.600 | 0.012 | 0.045 |
| k__Bacteria; p__Firmicutes; c__Clostridia; o__Clostridiales; f__Lachnospiraceae; g__; s__ | HA | 0.050 | 0.020 | 2.469 | 0.016 | 0.056 |
| k__Bacteria; p__Firmicutes; c__Bacilli; o__Bacillales; f__Staphylococcaceae; g__Staphylococcus; s__sciuri | HA | 1.177 | 0.478 | 2.461 | 0.017 | 0.056 |
| k__Bacteria; p__Firmicutes; c__Bacilli; o__Turicibacterales; f__Turicibacteraceae; g__Turicibacter; s__ | HA | 0.193 | 0.081 | 2.373 | 0.021 | 0.065 |
| k__Bacteria; p__Firmicutes; c__Bacilli; o__Lactobacillales; f__Lactobacillaceae; g__Lactobacillus; s__ | HA | 2.074 | 0.905 | 2.293 | 0.025 | 0.075 |
| k__Bacteria; p__Bacteroidetes; c__Bacteroidia; o__Bacteroidales; f__S24-7; g__; s__ | HA | 0.097 | 0.042 | 2.282 | 0.026 | 0.075 |
| k__Bacteria; p__Firmicutes; c__Clostridia; o__Clostridiales; f__; g__; s__ | HA | 0.085 | 0.038 | 2.225 | 0.030 | 0.084 |
| k__Bacteria; p__Firmicutes; c__Bacilli; o__Lactobacillales; f__Streptococcaceae; g__Streptococcus; s__ | HA | -0.337 | 0.157 | -2.150 | 0.035 | 0.096 |
| k__Bacteria; p__Firmicutes; c__Bacilli; o__Bacillales; f__Staphylococcaceae; g__Jeotgalicoccus; s__psychrophilus | HA | 0.365 | 0.180 | 2.027 | 0.047 | 0.117 |
| k__Bacteria; p__Firmicutes; c__Bacilli; o__Lactobacillales; f__Enterococcaceae; g__Enterococcus; s__ | HA | 0.759 | 0.399 | 1.904 | 0.061 | 0.136 |
| k__Bacteria; p__Firmicutes; c__Clostridia; o__Clostridiales; f__Peptostreptococcaceae; g__; s__ | HA | 0.171 | 0.090 | 1.899 | 0.062 | 0.136 |
| k__Bacteria; p__Firmicutes; c__Bacilli; o__Lactobacillales; f__Lactobacillaceae; g__Lactobacillus; s__ | HA | 0.069 | 0.038 | 1.819 | 0.074 | 0.159 |
| k__Bacteria; p__Bacteroidetes; c__Bacteroidia; o__Bacteroidales; f__S24-7; g__; s__ | HA | 0.136 | 0.086 | 1.583 | 0.118 | 0.224 |
| k__Bacteria; p__Firmicutes; c__Clostridia; o__Clostridiales; f__Ruminococcaceae; g__; s__ | HA | 0.042 | 0.028 | 1.521 | 0.133 | 0.240 |
| k__Bacteria; p__Firmicutes; c__Bacilli; o__Lactobacillales; f__Aerococcaceae; g__; s__ | HA | -0.058 | 0.042 | -1.385 | 0.171 | 0.292 |
| k__Bacteria; p__Firmicutes; c__Bacilli; o__Bacillales; f__Planococcaceae; g__Sporosarcina; s__ | HA | -0.048 | 0.038 | -1.263 | 0.211 | 0.326 |
| k__Bacteria; p__Firmicutes; c__Bacilli; o__Bacillales; f__Planococcaceae; g__Sporosarcina; s__ | HA | -0.063 | 0.051 | -1.229 | 0.224 | 0.334 |
| k__Bacteria; p__Firmicutes; c__Clostridia; o__Clostridiales; f__; g__; s__ | HA | 0.009 | 0.007 | 1.183 | 0.241 | 0.348 |
| k__Bacteria; p__Firmicutes; c__Bacilli; o__Bacillales; f__Planococcaceae; g__Sporosarcina; s__ | HA | -0.055 | 0.047 | -1.156 | 0.252 | 0.357 |
| k__Bacteria; p__Firmicutes; c__Erysipelotrichi; o__Erysipelotrichales; f__Erysipelotrichaceae; g__Allobaculum; s__ | HA | 0.101 | 0.105 | 0.963 | 0.339 | 0.402 |
| k__Bacteria; p__Actinobacteria; c__Coriobacteriia; o__Coriobacteriales; f__Coriobacteriaceae; g__Adlercreutzia; s__ | HA | 0.065 | 0.068 | 0.951 | 0.345 | 0.402 |
| k__Bacteria; p__Proteobacteria; c__Gammaproteobacteria; o__Enterobacteriales; f__Enterobacteriaceae; g__Pantoea; s__agglomerans | HA | 2.082 | 2.404 | 0.866 | 0.390 | 0.404 |
| k__Bacteria; p__Proteobacteria; c__Gammaproteobacteria; o__Enterobacteriales; f__Enterobacteriaceae; g__; s__ | HA | 0.070 | 0.081 | 0.856 | 0.395 | 0.405 |
| k__Bacteria; p__Proteobacteria; c__Gammaproteobacteria; o__Enterobacteriales; f__Enterobacteriaceae; g__; s__ | HA | -0.635 | 1.027 | -0.619 | 0.538 | 0.446 |
| k__Bacteria; p__Proteobacteria; c__Gammaproteobacteria; o__Enterobacteriales; f__Enterobacteriaceae; g__; s__ | HA | 0.042 | 0.085 | 0.496 | 0.622 | 0.465 |
| k__Bacteria; p__Firmicutes; c__Bacilli; o__Lactobacillales; f__Enterococcaceae; g__; s__ | HA | 0.004 | 0.059 | 0.071 | 0.944 | 0.531 |
| k__Bacteria; p__Firmicutes; c__Clostridia; o__Clostridiales; f__Lachnospiraceae; g__; s__ | GA | 1.240 | 0.408 | 3.039 | 0.004 | 0.020 |
| k__Bacteria; p__Firmicutes; c__Clostridia; o__Clostridiales; f__Lachnospiraceae; g__; s__ | GA | 0.478 | 0.172 | 2.780 | 0.007 | 0.033 |
| k__Bacteria; p__Firmicutes; c__Clostridia; o__Clostridiales; f__; g__; s__ | GA | 1.014 | 0.381 | 2.663 | 0.010 | 0.040 |
| k__Bacteria; p__Firmicutes; c__Erysipelotrichi; o__Erysipelotrichales; f__Erysipelotrichaceae; g__; s__ | GA | 1.354 | 0.515 | 2.628 | 0.011 | 0.043 |
| k__Bacteria; p__Actinobacteria; c__Coriobacteriia; o__Coriobacteriales; f__Coriobacteriaceae; g__Adlercreutzia; s__ | GA | 0.283 | 0.127 | 2.234 | 0.029 | 0.084 |
| k__Bacteria; p__Firmicutes; c__Clostridia; o__Clostridiales; f__Ruminococcaceae; g__; s__ | GA | 0.077 | 0.037 | 2.102 | 0.040 | 0.106 |
| k__Bacteria; p__Firmicutes; c__Clostridia; o__Clostridiales; f__; g__; s__ | GA | 0.294 | 0.143 | 2.056 | 0.044 | 0.114 |
| k__Bacteria; p__Verrucomicrobia; c__Verrucomicrobiae; o__Verrucomicrobiales; f__Verrucomicrobiaceae; g__Akkermansia; s__muciniphila | GA | 2.622 | 1.280 | 2.048 | 0.045 | 0.115 |
| k__Bacteria; p__Firmicutes; c__Bacilli; o__Bacillales; f__Planococcaceae; g__Sporosarcina; s__ | GA | -0.121 | 0.060 | -2.030 | 0.047 | 0.117 |
| k__Bacteria; p__Firmicutes; c__Bacilli; o__Lactobacillales; f__Aerococcaceae; g__; s__ | GA | -0.132 | 0.065 | -2.020 | 0.048 | 0.118 |
| k__Bacteria; p__Firmicutes; c__Bacilli; o__Bacillales; f__Planococcaceae; g__Sporosarcina; s__ | GA | -0.155 | 0.080 | -1.937 | 0.058 | 0.134 |
| k__Bacteria; p__Firmicutes; c__Bacilli; o__Bacillales; f__Planococcaceae; g__Sporosarcina; s__ | GA | -0.140 | 0.073 | -1.903 | 0.062 | 0.136 |
| k__Bacteria; p__Firmicutes; c__Clostridia; o__Clostridiales; f__; g__; s__ | GA | 0.102 | 0.055 | 1.848 | 0.070 | 0.151 |
| k__Bacteria; p__Firmicutes; c__Bacilli; o__Lactobacillales; f__Streptococcaceae; g__Streptococcus; s__ | GA | -0.376 | 0.227 | -1.654 | 0.104 | 0.201 |
| k__Bacteria; p__Firmicutes; c__Clostridia; o__Clostridiales; f__; g__; s__ | GA | 0.112 | 0.073 | 1.522 | 0.134 | 0.240 |
| k__Bacteria; p__Proteobacteria; c__Gammaproteobacteria; o__Enterobacteriales; f__Enterobacteriaceae; g__; s__ | GA | 2.415 | 1.608 | 1.502 | 0.139 | 0.247 |
| k__Bacteria; p__Proteobacteria; c__Gammaproteobacteria; o__Xanthomonadales; f__Xanthomonadaceae; g__Stenotrophomonas; s__ | GA | -0.037 | 0.027 | -1.362 | 0.178 | 0.302 |
| k__Bacteria; p__Firmicutes; c__Erysipelotrichi; o__Erysipelotrichales; f__Erysipelotrichaceae; g__Allobaculum; s__ | GA | 0.212 | 0.166 | 1.280 | 0.206 | 0.321 |
| k__Bacteria; p__Proteobacteria; c__Betaproteobacteria; o__Burkholderiales; f__Alcaligenaceae; g__Achromobacter; s__ | GA | -0.055 | 0.052 | -1.070 | 0.289 | 0.387 |
| k__Bacteria; p__Actinobacteria; c__Coriobacteriia; o__Coriobacteriales; f__Coriobacteriaceae; g__Adlercreutzia; s__ | GA | -0.117 | 0.113 | -1.042 | 0.302 | 0.392 |
| k__Bacteria; p__Proteobacteria; c__Gammaproteobacteria; o__Enterobacteriales; f__Enterobacteriaceae; g__; s__ | GA | 0.019 | 0.020 | 0.985 | 0.329 | 0.402 |
| k__Bacteria; p__Bacteroidetes; c__Bacteroidia; o__Bacteroidales; f__S24-7; g__; s__ | GA | 0.131 | 0.136 | 0.967 | 0.337 | 0.402 |
| k__Bacteria; p__Proteobacteria; c__Gammaproteobacteria; o__Alteromonadales; f__Shewanellaceae; g__Shewanella; s__algae | GA | -0.054 | 0.058 | -0.934 | 0.354 | 0.402 |
| k__Bacteria; p__Firmicutes; c__Bacilli; o__Lactobacillales; f__Enterococcaceae; g__; s__ | GA | -0.131 | 0.154 | -0.850 | 0.399 | 0.407 |
| k__Bacteria; p__Firmicutes; c__Clostridia; o__Clostridiales; f__Lachnospiraceae; g__Dorea; s__ | GA | 0.077 | 0.101 | 0.763 | 0.449 | 0.423 |
| k__Bacteria; p__Proteobacteria; c__Alphaproteobacteria; o__Caulobacterales; f__Caulobacteraceae; g__; s__ | GA | -0.026 | 0.034 | -0.746 | 0.459 | 0.427 |
| k__Bacteria; p__Firmicutes; c__Clostridia; o__Clostridiales; f__Peptostreptococcaceae; g__; s__ | GA | -0.109 | 0.146 | -0.742 | 0.461 | 0.427 |
| k__Bacteria; p__Firmicutes; c__Bacilli; o__Bacillales; f__Staphylococcaceae; g__Staphylococcus; s__succinus | GA | -0.355 | 0.487 | -0.728 | 0.470 | 0.429 |
| k__Bacteria; p__Actinobacteria; c__Coriobacteriia; o__Coriobacteriales; f__Coriobacteriaceae; g__; s__ | GA | 1.446 | 2.026 | 0.714 | 0.478 | 0.432 |
| k__Bacteria; p__Proteobacteria; c__Gammaproteobacteria; o__Pseudomonadales; f__Pseudomonadaceae; g__Pseudomonas; s__veronii | GA | -2.438 | 3.518 | -0.693 | 0.491 | 0.438 |
| k__Bacteria; p__Actinobacteria; c__Coriobacteriia; o__Coriobacteriales; f__Coriobacteriaceae; g__Adlercreutzia; s__ | GA | 0.062 | 0.090 | 0.687 | 0.495 | 0.439 |
| k__Bacteria; p__Firmicutes; c__Bacilli; o__Lactobacillales; f__Lactobacillaceae; g__Lactobacillus; s__ | GA | -1.353 | 2.026 | -0.668 | 0.507 | 0.445 |
| k__Bacteria; p__Proteobacteria; c__Gammaproteobacteria; o__Xanthomonadales; f__Sinobacteraceae; g__Nevskia; s__ | GA | -0.062 | 0.094 | -0.656 | 0.514 | 0.445 |
| k__Bacteria; p__Proteobacteria; c__Gammaproteobacteria; o__Pseudomonadales; f__Pseudomonadaceae; g__Pseudomonas; s__ | GA | -0.050 | 0.078 | -0.646 | 0.521 | 0.445 |
| k__Bacteria; p__Bacteroidetes; c__[Saprospirae]; o__[Saprospirales]; f__Chitinophagaceae; g__Sediminibacterium; s__ | GA | -0.010 | 0.016 | -0.623 | 0.536 | 0.446 |
| k__Bacteria; p__Bacteroidetes; c__Bacteroidia; o__Bacteroidales; f__S24-7; g__; s__ | GA | 0.036 | 0.060 | 0.604 | 0.548 | 0.446 |
| k__Bacteria; p__Firmicutes; c__Bacilli; o__Lactobacillales; f__Lactobacillaceae; g__Lactobacillus; s__ | GA | -0.040 | 0.066 | -0.601 | 0.550 | 0.446 |
| k__Bacteria; p__Firmicutes; c__Bacilli; o__Lactobacillales; f__Lactobacillaceae; g__Lactobacillus; s__ | GA | -1.046 | 1.781 | -0.588 | 0.559 | 0.446 |
| k__Bacteria; p__Proteobacteria; c__Gammaproteobacteria; o__Pseudomonadales; f__Pseudomonadaceae; g__Pseudomonas; s__ | GA | -0.024 | 0.040 | -0.587 | 0.560 | 0.446 |
| k__Bacteria; p__Firmicutes; c__Bacilli; o__Turicibacterales; f__Turicibacteraceae; g__Turicibacter; s__ | GA | -0.079 | 0.134 | -0.586 | 0.560 | 0.446 |
| k__Bacteria; p__Firmicutes; c__Bacilli; o__Lactobacillales; f__Lactobacillaceae; g__Lactobacillus; s__ | GA | -0.058 | 0.102 | -0.562 | 0.577 | 0.454 |
| k__Bacteria; p__Proteobacteria; c__Gammaproteobacteria; o__Enterobacteriales; f__Enterobacteriaceae; g__; s__ | GA | 0.005 | 0.009 | 0.556 | 0.580 | 0.454 |
| k__Bacteria; p__Proteobacteria; c__Gammaproteobacteria; o__Pseudomonadales; f__Pseudomonadaceae; g__Pseudomonas; s__veronii | GA | -0.016 | 0.030 | -0.524 | 0.602 | 0.460 |
| k__Bacteria; p__Bacteroidetes; c__Bacteroidia; o__Bacteroidales; f__S24-7; g__; s__ | GA | 1.081 | 2.201 | 0.491 | 0.625 | 0.465 |
| k__Bacteria; p__Proteobacteria; c__Gammaproteobacteria; o__Enterobacteriales; f__Enterobacteriaceae; g__Pantoea; s__agglomerans | GA | 0.109 | 0.245 | 0.447 | 0.657 | 0.471 |
| k__Bacteria; p__Firmicutes; c__Clostridia; o__Clostridiales; f__Lachnospiraceae; g__; s__ | GA | 0.116 | 0.261 | 0.445 | 0.658 | 0.471 |
| k__Bacteria; p__Firmicutes; c__Bacilli; o__Lactobacillales; f__Enterococcaceae; g__Enterococcus; s__ | GA | -0.262 | 0.647 | -0.404 | 0.687 | 0.471 |
| k__Bacteria; p__Firmicutes; c__Clostridia; o__Clostridiales; f__; g__; s__ | GA | -0.053 | 0.133 | -0.403 | 0.689 | 0.471 |
| k__Bacteria; p__Firmicutes; c__Clostridia; o__Clostridiales; f__Ruminococcaceae; g__; s__ | GA | 0.025 | 0.063 | 0.399 | 0.692 | 0.471 |
| k__Bacteria; p__Firmicutes; c__Clostridia; o__Clostridiales; f__Ruminococcaceae; g__; s__ | GA | 0.066 | 0.192 | 0.345 | 0.731 | 0.478 |
| k__Bacteria; p__Proteobacteria; c__Gammaproteobacteria; o__Oceanospirillales; f__Halomonadaceae; g__Halomonas; s__ | GA | -0.023 | 0.072 | -0.325 | 0.747 | 0.482 |
| k__Bacteria; p__Firmicutes; c__Clostridia; o__Clostridiales; f__; g__; s__ | GA | 0.042 | 0.135 | 0.309 | 0.758 | 0.483 |
| k__Bacteria; p__Actinobacteria; c__Actinobacteria; o__Bifidobacteriales; f__Bifidobacteriaceae; g__Bifidobacterium; s__ | GA | -0.026 | 0.086 | -0.304 | 0.762 | 0.483 |
| k__Bacteria; p__Actinobacteria; c__Actinobacteria; o__Bifidobacteriales; f__Bifidobacteriaceae; g__Bifidobacterium; s__pseudolongum | GA | -1.716 | 5.938 | -0.289 | 0.774 | 0.488 |
| k__Bacteria; p__Firmicutes; c__Bacilli; o__Lactobacillales; f__Aerococcaceae; g__Aerococcus; s__ | GA | 0.059 | 0.253 | 0.231 | 0.818 | 0.503 |
| k__Bacteria; p__Firmicutes; c__Bacilli; o__Bacillales; f__Staphylococcaceae; g__Jeotgalicoccus; s__psychrophilus | GA | 0.038 | 0.296 | 0.130 | 0.897 | 0.518 |
| k__Bacteria; p__Firmicutes; c__Bacilli; o__Bacillales; f__Staphylococcaceae; g__Staphylococcus; s__sciuri | GA | -0.094 | 0.797 | -0.119 | 0.906 | 0.522 |
| k__Bacteria; p__Bacteroidetes; c__Bacteroidia; o__Bacteroidales; f__S24-7; g__; s__ | GA | 0.005 | 0.050 | 0.090 | 0.928 | 0.528 |
| k__Bacteria; p__Proteobacteria; c__Alphaproteobacteria; o__Rhizobiales; f__Bradyrhizobiaceae; g__Bradyrhizobium; s__ | GA | 0.002 | 0.025 | 0.089 | 0.930 | 0.528 |
| k__Bacteria; p__Bacteroidetes; c__Bacteroidia; o__Bacteroidales; f__S24-7; g__; s__ | GA | 0.101 | 1.530 | 0.066 | 0.948 | 0.531 |
| k__Bacteria; p__Proteobacteria; c__Gammaproteobacteria; o__Oceanospirillales; f__Halomonadaceae; g__Halomonas; s__ | GA | -0.003 | 0.053 | -0.064 | 0.949 | 0.531 |
| k__Bacteria; p__Firmicutes; c__Clostridia; o__Clostridiales; f__Clostridiaceae; g__; s__ | GA | 0.002 | 0.062 | 0.035 | 0.972 | 0.538 |
| k__Bacteria; p__Firmicutes; c__Bacilli; o__Lactobacillales; f__Aerococcaceae; g__; s__ | FA | -1.985 | 0.676 | -2.936 | 0.005 | 0.023 |
| k__Bacteria; p__Firmicutes; c__Bacilli; o__Bacillales; f__Planococcaceae; g__Sporosarcina; s__ | FA | -2.242 | 0.826 | -2.713 | 0.008 | 0.035 |
| k__Bacteria; p__Bacteroidetes; c__Bacteroidia; o__Bacteroidales; f__S24-7; g__; s__ | FA | 3.569 | 1.439 | 2.481 | 0.016 | 0.055 |
| k__Bacteria; p__Firmicutes; c__Bacilli; o__Bacillales; f__Planococcaceae; g__Sporosarcina; s__ | FA | -1.501 | 0.634 | -2.366 | 0.021 | 0.065 |
| k__Bacteria; p__Firmicutes; c__Clostridia; o__Clostridiales; f__; g__; s__ | FA | 1.748 | 0.746 | 2.342 | 0.022 | 0.068 |
| k__Bacteria; p__Firmicutes; c__Bacilli; o__Bacillales; f__Planococcaceae; g__Sporosarcina; s__ | FA | -1.736 | 0.789 | -2.201 | 0.031 | 0.088 |
| k__Bacteria; p__Bacteroidetes; c__Bacteroidia; o__Bacteroidales; f__S24-7; g__; s__ | FA | 1.308 | 0.654 | 2.001 | 0.050 | 0.120 |
| k__Bacteria; p__Firmicutes; c__Clostridia; o__Clostridiales; f__; g__; s__ | FA | 2.373 | 1.205 | 1.969 | 0.053 | 0.127 |
| k__Bacteria; p__Firmicutes; c__Erysipelotrichi; o__Erysipelotrichales; f__Erysipelotrichaceae; g__Allobaculum; s__ | FA | 3.364 | 1.760 | 1.911 | 0.060 | 0.136 |
| k__Bacteria; p__Firmicutes; c__Bacilli; o__Lactobacillales; f__Enterococcaceae; g__; s__ | FA | -2.872 | 1.627 | -1.765 | 0.082 | 0.175 |
| k__Bacteria; p__Firmicutes; c__Bacilli; o__Lactobacillales; f__Lactobacillaceae; g__Lactobacillus; s__ | FA | -37.941 | 21.631 | -1.754 | 0.084 | 0.178 |
| k__Bacteria; p__Firmicutes; c__Clostridia; o__Clostridiales; f__Clostridiaceae; g__; s__ | FA | 1.080 | 0.629 | 1.719 | 0.090 | 0.185 |
| k__Bacteria; p__Actinobacteria; c__Coriobacteriia; o__Coriobacteriales; f__Coriobacteriaceae; g__Adlercreutzia; s__ | FA | -1.900 | 1.223 | -1.554 | 0.125 | 0.234 |
| k__Bacteria; p__Firmicutes; c__Clostridia; o__Clostridiales; f__; g__; s__ | FA | 0.963 | 0.657 | 1.465 | 0.148 | 0.258 |
| k__Bacteria; p__Bacteroidetes; c__Bacteroidia; o__Bacteroidales; f__S24-7; g__; s__ | FA | 35.840 | 25.138 | 1.426 | 0.159 | 0.276 |
| k__Bacteria; p__Firmicutes; c__Bacilli; o__Lactobacillales; f__Lactobacillaceae; g__Lactobacillus; s__ | FA | -0.981 | 0.710 | -1.381 | 0.172 | 0.292 |
| k__Bacteria; p__Firmicutes; c__Bacilli; o__Bacillales; f__Staphylococcaceae; g__Staphylococcus; s__succinus | FA | -7.062 | 5.210 | -1.355 | 0.180 | 0.302 |
| k__Bacteria; p__Bacteroidetes; c__Bacteroidia; o__Bacteroidales; f__S24-7; g__; s__ | FA | 23.114 | 17.244 | 1.340 | 0.185 | 0.309 |
| k__Bacteria; p__Firmicutes; c__Clostridia; o__Clostridiales; f__Ruminococcaceae; g__; s__ | FA | 0.751 | 0.565 | 1.328 | 0.189 | 0.310 |
| k__Bacteria; p__Verrucomicrobia; c__Verrucomicrobiae; o__Verrucomicrobiales; f__Verrucomicrobiaceae; g__Akkermansia; s__muciniphila | FA | 19.712 | 14.908 | 1.322 | 0.191 | 0.312 |
| k__Bacteria; p__Firmicutes; c__Clostridia; o__Clostridiales; f__Ruminococcaceae; g__; s__ | FA | 2.812 | 2.143 | 1.312 | 0.194 | 0.316 |
| k__Bacteria; p__Firmicutes; c__Clostridia; o__Clostridiales; f__; g__; s__ | FA | 0.676 | 0.524 | 1.289 | 0.202 | 0.318 |
| k__Bacteria; p__Firmicutes; c__Bacilli; o__Turicibacterales; f__Turicibacteraceae; g__Turicibacter; s__ | FA | 1.759 | 1.436 | 1.225 | 0.225 | 0.334 |
| k__Bacteria; p__Firmicutes; c__Bacilli; o__Lactobacillales; f__Lactobacillaceae; g__Lactobacillus; s__ | FA | -1.213 | 1.113 | -1.089 | 0.280 | 0.379 |
| k__Bacteria; p__Proteobacteria; c__Gammaproteobacteria; o__Alteromonadales; f__Shewanellaceae; g__Shewanella; s__algae | FA | -0.697 | 0.658 | -1.059 | 0.293 | 0.388 |
| k__Bacteria; p__Firmicutes; c__Clostridia; o__Clostridiales; f__Lachnospiraceae; g__; s__ | FA | 3.023 | 2.891 | 1.045 | 0.300 | 0.392 |
| k__Bacteria; p__Firmicutes; c__Bacilli; o__Lactobacillales; f__Lactobacillaceae; g__Lactobacillus; s__ | FA | -19.737 | 19.455 | -1.015 | 0.314 | 0.399 |
| k__Bacteria; p__Actinobacteria; c__Actinobacteria; o__Bifidobacteriales; f__Bifidobacteriaceae; g__Bifidobacterium; s__ | FA | -0.919 | 0.929 | -0.989 | 0.326 | 0.402 |
| k__Bacteria; p__Firmicutes; c__Clostridia; o__Clostridiales; f__Ruminococcaceae; g__; s__ | FA | 0.588 | 0.600 | 0.979 | 0.331 | 0.402 |
| k__Bacteria; p__Proteobacteria; c__Betaproteobacteria; o__Burkholderiales; f__Alcaligenaceae; g__Achromobacter; s__ | FA | -0.568 | 0.581 | -0.978 | 0.332 | 0.402 |
| k__Bacteria; p__Actinobacteria; c__Actinobacteria; o__Bifidobacteriales; f__Bifidobacteriaceae; g__Bifidobacterium; s__pseudolongum | FA | -60.883 | 64.150 | -0.949 | 0.346 | 0.402 |
| k__Bacteria; p__Firmicutes; c__Clostridia; o__Clostridiales; f__; g__; s__ | FA | 1.451 | 1.587 | 0.914 | 0.364 | 0.402 |
| k__Bacteria; p__Actinobacteria; c__Coriobacteriia; o__Coriobacteriales; f__Coriobacteriaceae; g__Adlercreutzia; s__ | FA | 1.274 | 1.421 | 0.897 | 0.373 | 0.402 |
| k__Bacteria; p__Firmicutes; c__Erysipelotrichi; o__Erysipelotrichales; f__Erysipelotrichaceae; g__; s__ | FA | 5.150 | 5.891 | 0.874 | 0.385 | 0.403 |
| k__Bacteria; p__Proteobacteria; c__Gammaproteobacteria; o__Xanthomonadales; f__Xanthomonadaceae; g__Stenotrophomonas; s__ | FA | -0.267 | 0.308 | -0.867 | 0.389 | 0.404 |
| k__Bacteria; p__Firmicutes; c__Clostridia; o__Clostridiales; f__; g__; s__ | FA | 3.472 | 4.301 | 0.807 | 0.422 | 0.409 |
| k__Bacteria; p__Firmicutes; c__Clostridia; o__Clostridiales; f__Lachnospiraceae; g__; s__ | FA | 3.743 | 4.709 | 0.795 | 0.430 | 0.409 |
| k__Bacteria; p__Proteobacteria; c__Gammaproteobacteria; o__Enterobacteriales; f__Enterobacteriaceae; g__; s__ | FA | 13.107 | 17.561 | 0.746 | 0.458 | 0.427 |
| k__Bacteria; p__Firmicutes; c__Clostridia; o__Clostridiales; f__Lachnospiraceae; g__; s__ | FA | 1.419 | 1.944 | 0.730 | 0.468 | 0.429 |
| k__Bacteria; p__Proteobacteria; c__Alphaproteobacteria; o__Rhizobiales; f__Bradyrhizobiaceae; g__Bradyrhizobium; s__ | FA | 0.165 | 0.276 | 0.597 | 0.553 | 0.446 |
| k__Bacteria; p__Proteobacteria; c__Gammaproteobacteria; o__Pseudomonadales; f__Pseudomonadaceae; g__Pseudomonas; s__ | FA | -0.525 | 0.895 | -0.587 | 0.560 | 0.446 |
| k__Bacteria; p__Proteobacteria; c__Gammaproteobacteria; o__Pseudomonadales; f__Pseudomonadaceae; g__Pseudomonas; s__ | FA | -0.260 | 0.466 | -0.557 | 0.580 | 0.454 |
| k__Bacteria; p__Actinobacteria; c__Coriobacteriia; o__Coriobacteriales; f__Coriobacteriaceae; g__; s__ | FA | -12.030 | 21.805 | -0.552 | 0.583 | 0.454 |
| k__Bacteria; p__Firmicutes; c__Bacilli; o__Bacillales; f__Staphylococcaceae; g__Jeotgalicoccus; s__psychrophilus | FA | 1.744 | 3.179 | 0.549 | 0.585 | 0.454 |
| k__Bacteria; p__Proteobacteria; c__Gammaproteobacteria; o__Pseudomonadales; f__Pseudomonadaceae; g__Pseudomonas; s__veronii | FA | -0.169 | 0.348 | -0.486 | 0.629 | 0.465 |
| k__Bacteria; p__Firmicutes; c__Clostridia; o__Clostridiales; f__Lachnospiraceae; g__Dorea; s__ | FA | -0.462 | 1.082 | -0.427 | 0.671 | 0.471 |
| k__Bacteria; p__Actinobacteria; c__Coriobacteriia; o__Coriobacteriales; f__Coriobacteriaceae; g__Adlercreutzia; s__ | FA | 0.402 | 0.987 | 0.407 | 0.685 | 0.471 |
| k__Bacteria; p__Proteobacteria; c__Gammaproteobacteria; o__Pseudomonadales; f__Pseudomonadaceae; g__Pseudomonas; s__veronii | FA | -15.155 | 40.551 | -0.374 | 0.710 | 0.475 |
| k__Bacteria; p__Proteobacteria; c__Gammaproteobacteria; o__Oceanospirillales; f__Halomonadaceae; g__Halomonas; s__ | FA | -0.303 | 0.826 | -0.366 | 0.715 | 0.475 |
| k__Bacteria; p__Firmicutes; c__Bacilli; o__Lactobacillales; f__Enterococcaceae; g__Enterococcus; s__ | FA | -2.530 | 7.007 | -0.361 | 0.719 | 0.476 |
| k__Bacteria; p__Proteobacteria; c__Alphaproteobacteria; o__Caulobacterales; f__Caulobacteraceae; g__; s__ | FA | -0.134 | 0.396 | -0.337 | 0.737 | 0.479 |
| k__Bacteria; p__Proteobacteria; c__Gammaproteobacteria; o__Enterobacteriales; f__Enterobacteriaceae; g__; s__ | FA | 0.477 | 1.457 | 0.327 | 0.745 | 0.482 |
| k__Bacteria; p__Firmicutes; c__Bacilli; o__Lactobacillales; f__Aerococcaceae; g__Aerococcus; s__ | FA | 0.860 | 2.726 | 0.316 | 0.753 | 0.483 |
| k__Bacteria; p__Bacteroidetes; c__[Saprospirae]; o__[Saprospirales]; f__Chitinophagaceae; g__Sediminibacterium; s__ | FA | 0.060 | 0.190 | 0.314 | 0.755 | 0.483 |
| k__Bacteria; p__Firmicutes; c__Bacilli; o__Lactobacillales; f__Streptococcaceae; g__Streptococcus; s__ | FA | -0.665 | 2.784 | -0.239 | 0.812 | 0.500 |
| k__Bacteria; p__Bacteroidetes; c__Bacteroidia; o__Bacteroidales; f__S24-7; g__; s__ | FA | -0.196 | 0.855 | -0.230 | 0.819 | 0.503 |
| k__Bacteria; p__Firmicutes; c__Clostridia; o__Clostridiales; f__Peptostreptococcaceae; g__; s__ | FA | -0.318 | 1.433 | -0.222 | 0.825 | 0.504 |
| k__Bacteria; p__Proteobacteria; c__Gammaproteobacteria; o__Enterobacteriales; f__Enterobacteriaceae; g__; s__ | FA | 0.274 | 1.403 | 0.196 | 0.846 | 0.512 |
| k__Bacteria; p__Proteobacteria; c__Gammaproteobacteria; o__Enterobacteriales; f__Enterobacteriaceae; g__Pantoea; s__agglomerans | FA | 7.964 | 41.398 | 0.192 | 0.848 | 0.512 |
| k__Bacteria; p__Proteobacteria; c__Gammaproteobacteria; o__Xanthomonadales; f__Sinobacteraceae; g__Nevskia; s__ | FA | -0.203 | 1.086 | -0.187 | 0.852 | 0.512 |
| k__Bacteria; p__Proteobacteria; c__Gammaproteobacteria; o__Oceanospirillales; f__Halomonadaceae; g__Halomonas; s__ | FA | 0.088 | 0.593 | 0.149 | 0.882 | 0.518 |
| k__Bacteria; p__Firmicutes; c__Bacilli; o__Bacillales; f__Staphylococcaceae; g__Staphylococcus; s__sciuri | FA | 0.115 | 8.480 | 0.014 | 0.989 | 0.540 |
| k__Bacteria; p__Firmicutes; c__Clostridia; o__Clostridiales; f__; g__; s__ | 4-HPVA | 0.124 | 0.014 | 8.783 | 0.000 | 0.000 |
| k__Bacteria; p__Firmicutes; c__Clostridia; o__Clostridiales; f__Lachnospiraceae; g__Dorea; s__ | 4-HPVA | 2.077 | 0.270 | 7.696 | 0.000 | 0.000 |
| k__Bacteria; p__Firmicutes; c__Clostridia; o__Clostridiales; f__; g__; s__ | 4-HPVA | 0.789 | 0.105 | 7.514 | 0.000 | 0.000 |
| k__Bacteria; p__Firmicutes; c__Clostridia; o__Clostridiales; f__Lachnospiraceae; g__; s__ | 4-HPVA | 1.552 | 0.236 | 6.586 | 0.000 | 0.000 |
| k__Bacteria; p__Firmicutes; c__Clostridia; o__Clostridiales; f__Ruminococcaceae; g__; s__ | 4-HPVA | 1.026 | 0.168 | 6.112 | 0.000 | 0.000 |
| k__Bacteria; p__Firmicutes; c__Clostridia; o__Clostridiales; f__; g__; s__ | 4-HPVA | 1.011 | 0.169 | 5.995 | 0.000 | 0.000 |
| k__Bacteria; p__Firmicutes; c__Clostridia; o__Clostridiales; f__Lachnospiraceae; g__; s__ | 4-HPVA | 0.818 | 0.138 | 5.926 | 0.000 | 0.000 |
| k__Bacteria; p__Firmicutes; c__Clostridia; o__Clostridiales; f__Ruminococcaceae; g__; s__ | 4-HPVA | 0.236 | 0.045 | 5.284 | 0.000 | 0.000 |
| k__Bacteria; p__Firmicutes; c__Clostridia; o__Clostridiales; f__; g__; s__ | 4-HPVA | 2.160 | 0.474 | 4.556 | 0.000 | 0.000 |
| k__Bacteria; p__Verrucomicrobia; c__Verrucomicrobiae; o__Verrucomicrobiales; f__Verrucomicrobiaceae; g__Akkermansia; s__muciniphila | 4-HPVA | 10.621 | 2.617 | 4.058 | 0.000 | 0.001 |
| k__Bacteria; p__Actinobacteria; c__Coriobacteriia; o__Coriobacteriales; f__Coriobacteriaceae; g__Adlercreutzia; s__ | 4-HPVA | 1.001 | 0.265 | 3.773 | 0.000 | 0.003 |
| k__Bacteria; p__Firmicutes; c__Erysipelotrichi; o__Erysipelotrichales; f__Erysipelotrichaceae; g__; s__ | 4-HPVA | 1.918 | 0.513 | 3.742 | 0.000 | 0.003 |
| k__Bacteria; p__Bacteroidetes; c__Bacteroidia; o__Bacteroidales; f__S24-7; g__; s__ | 4-HPVA | 29.833 | 8.561 | 3.485 | 0.001 | 0.006 |
| k__Bacteria; p__Firmicutes; c__Clostridia; o__Clostridiales; f__; g__; s__ | 4-HPVA | 1.552 | 0.539 | 2.880 | 0.005 | 0.026 |
| k__Bacteria; p__Firmicutes; c__Clostridia; o__Clostridiales; f__Clostridiaceae; g__; s__ | 4-HPVA | 0.678 | 0.250 | 2.712 | 0.008 | 0.035 |
| k__Bacteria; p__Actinobacteria; c__Coriobacteriia; o__Coriobacteriales; f__Coriobacteriaceae; g__Adlercreutzia; s__ | 4-HPVA | 0.779 | 0.294 | 2.646 | 0.010 | 0.040 |
| k__Bacteria; p__Bacteroidetes; c__Bacteroidia; o__Bacteroidales; f__S24-7; g__; s__ | 4-HPVA | 9.287 | 3.649 | 2.545 | 0.013 | 0.049 |
| k__Bacteria; p__Proteobacteria; c__Gammaproteobacteria; o__Xanthomonadales; f__Sinobacteraceae; g__Nevskia; s__ | 4-HPVA | -1.009 | 0.400 | -2.524 | 0.014 | 0.051 |
| k__Bacteria; p__Proteobacteria; c__Gammaproteobacteria; o__Pseudomonadales; f__Pseudomonadaceae; g__Pseudomonas; s__veronii | 4-HPVA | -37.539 | 15.081 | -2.489 | 0.015 | 0.055 |
| k__Bacteria; p__Proteobacteria; c__Betaproteobacteria; o__Burkholderiales; f__Alcaligenaceae; g__Achromobacter; s__ | 4-HPVA | -0.539 | 0.217 | -2.480 | 0.016 | 0.055 |
| k__Bacteria; p__Firmicutes; c__Clostridia; o__Clostridiales; f__Peptostreptococcaceae; g__; s__ | 4-HPVA | 1.413 | 0.574 | 2.462 | 0.016 | 0.056 |
| k__Bacteria; p__Proteobacteria; c__Gammaproteobacteria; o__Alteromonadales; f__Shewanellaceae; g__Shewanella; s__algae | 4-HPVA | -0.599 | 0.245 | -2.442 | 0.017 | 0.058 |
| k__Bacteria; p__Proteobacteria; c__Alphaproteobacteria; o__Rhizobiales; f__Bradyrhizobiaceae; g__Bradyrhizobium; s__ | 4-HPVA | -0.246 | 0.102 | -2.405 | 0.019 | 0.063 |
| k__Bacteria; p__Proteobacteria; c__Gammaproteobacteria; o__Oceanospirillales; f__Halomonadaceae; g__Halomonas; s__ | 4-HPVA | -0.728 | 0.304 | -2.390 | 0.020 | 0.064 |
| k__Bacteria; p__Proteobacteria; c__Gammaproteobacteria; o__Pseudomonadales; f__Pseudomonadaceae; g__Pseudomonas; s__ | 4-HPVA | -0.413 | 0.173 | -2.384 | 0.020 | 0.065 |
| k__Bacteria; p__Proteobacteria; c__Gammaproteobacteria; o__Xanthomonadales; f__Xanthomonadaceae; g__Stenotrophomonas; s__ | 4-HPVA | -0.281 | 0.118 | -2.376 | 0.020 | 0.065 |
| k__Bacteria; p__Proteobacteria; c__Alphaproteobacteria; o__Caulobacterales; f__Caulobacteraceae; g__; s__ | 4-HPVA | -0.351 | 0.148 | -2.371 | 0.021 | 0.065 |
| k__Bacteria; p__Bacteroidetes; c__[Saprospirae]; o__[Saprospirales]; f__Chitinophagaceae; g__Sediminibacterium; s__ | 4-HPVA | -0.165 | 0.071 | -2.329 | 0.023 | 0.070 |
| k__Bacteria; p__Proteobacteria; c__Gammaproteobacteria; o__Pseudomonadales; f__Pseudomonadaceae; g__Pseudomonas; s__veronii | 4-HPVA | -0.296 | 0.129 | -2.287 | 0.025 | 0.075 |
| k__Bacteria; p__Proteobacteria; c__Gammaproteobacteria; o__Pseudomonadales; f__Pseudomonadaceae; g__Pseudomonas; s__ | 4-HPVA | -0.737 | 0.331 | -2.225 | 0.029 | 0.084 |
| k__Bacteria; p__Proteobacteria; c__Gammaproteobacteria; o__Oceanospirillales; f__Halomonadaceae; g__Halomonas; s__ | 4-HPVA | -0.481 | 0.222 | -2.166 | 0.034 | 0.093 |
| k__Bacteria; p__Firmicutes; c__Clostridia; o__Clostridiales; f__Ruminococcaceae; g__; s__ | 4-HPVA | 1.736 | 0.830 | 2.092 | 0.040 | 0.106 |
| k__Bacteria; p__Actinobacteria; c__Coriobacteriia; o__Coriobacteriales; f__Coriobacteriaceae; g__; s__ | 4-HPVA | 15.906 | 9.118 | 1.744 | 0.086 | 0.180 |
| k__Bacteria; p__Bacteroidetes; c__Bacteroidia; o__Bacteroidales; f__S24-7; g__; s__ | 4-HPVA | 0.299 | 0.176 | 1.699 | 0.094 | 0.191 |
| k__Bacteria; p__Firmicutes; c__Clostridia; o__Clostridiales; f__Lachnospiraceae; g__; s__ | 4-HPVA | 0.147 | 0.099 | 1.485 | 0.142 | 0.250 |
| k__Bacteria; p__Firmicutes; c__Bacilli; o__Lactobacillales; f__Streptococcaceae; g__Streptococcus; s__ | 4-HPVA | -1.042 | 1.049 | -0.994 | 0.324 | 0.402 |
| k__Bacteria; p__Bacteroidetes; c__Bacteroidia; o__Bacteroidales; f__S24-7; g__; s__ | 4-HPVA | 0.171 | 0.230 | 0.741 | 0.461 | 0.427 |
| k__Bacteria; p__Actinobacteria; c__Actinobacteria; o__Bifidobacteriales; f__Bifidobacteriaceae; g__Bifidobacterium; s__pseudolongum | 4-HPVA | 18.009 | 24.392 | 0.738 | 0.463 | 0.427 |
| k__Bacteria; p__Actinobacteria; c__Actinobacteria; o__Bifidobacteriales; f__Bifidobacteriaceae; g__Bifidobacterium; s__ | 4-HPVA | 0.247 | 0.354 | 0.699 | 0.487 | 0.436 |
| k__Bacteria; p__Firmicutes; c__Bacilli; o__Lactobacillales; f__Aerococcaceae; g__; s__ | 4-HPVA | -0.186 | 0.276 | -0.675 | 0.502 | 0.442 |
| k__Bacteria; p__Firmicutes; c__Bacilli; o__Bacillales; f__Planococcaceae; g__Sporosarcina; s__ | 4-HPVA | -0.167 | 0.252 | -0.661 | 0.511 | 0.445 |
| k__Bacteria; p__Firmicutes; c__Bacilli; o__Bacillales; f__Planococcaceae; g__Sporosarcina; s__ | 4-HPVA | -0.191 | 0.309 | -0.620 | 0.537 | 0.446 |
| k__Bacteria; p__Firmicutes; c__Bacilli; o__Bacillales; f__Planococcaceae; g__Sporosarcina; s__ | 4-HPVA | -0.200 | 0.337 | -0.593 | 0.555 | 0.446 |
| k__Bacteria; p__Firmicutes; c__Bacilli; o__Lactobacillales; f__Aerococcaceae; g__Aerococcus; s__ | 4-HPVA | -0.578 | 1.134 | -0.510 | 0.612 | 0.463 |
| k__Bacteria; p__Proteobacteria; c__Gammaproteobacteria; o__Enterobacteriales; f__Enterobacteriaceae; g__; s__ | 4-HPVA | -0.253 | 0.551 | -0.459 | 0.648 | 0.471 |
| k__Bacteria; p__Firmicutes; c__Bacilli; o__Turicibacterales; f__Turicibacteraceae; g__Turicibacter; s__ | 4-HPVA | -0.229 | 0.549 | -0.418 | 0.677 | 0.471 |
| k__Bacteria; p__Firmicutes; c__Bacilli; o__Bacillales; f__Staphylococcaceae; g__Staphylococcus; s__sciuri | 4-HPVA | 1.312 | 3.252 | 0.403 | 0.688 | 0.471 |
| k__Bacteria; p__Bacteroidetes; c__Bacteroidia; o__Bacteroidales; f__S24-7; g__; s__ | 4-HPVA | -0.224 | 0.567 | -0.394 | 0.694 | 0.471 |
| k__Bacteria; p__Firmicutes; c__Bacilli; o__Bacillales; f__Staphylococcaceae; g__Staphylococcus; s__succinus | 4-HPVA | -0.658 | 1.997 | -0.329 | 0.743 | 0.482 |
| k__Bacteria; p__Firmicutes; c__Bacilli; o__Lactobacillales; f__Lactobacillaceae; g__Lactobacillus; s__ | 4-HPVA | -0.085 | 0.274 | -0.311 | 0.757 | 0.483 |
| k__Bacteria; p__Proteobacteria; c__Gammaproteobacteria; o__Enterobacteriales; f__Enterobacteriaceae; g__; s__ | 4-HPVA | -0.144 | 0.531 | -0.271 | 0.787 | 0.495 |
| k__Bacteria; p__Proteobacteria; c__Gammaproteobacteria; o__Enterobacteriales; f__Enterobacteriaceae; g__Pantoea; s__agglomerans | 4-HPVA | -4.117 | 15.672 | -0.263 | 0.794 | 0.497 |
| k__Bacteria; p__Firmicutes; c__Bacilli; o__Bacillales; f__Staphylococcaceae; g__Jeotgalicoccus; s__psychrophilus | 4-HPVA | -0.302 | 1.205 | -0.251 | 0.803 | 0.499 |
| k__Bacteria; p__Firmicutes; c__Bacilli; o__Lactobacillales; f__Lactobacillaceae; g__Lactobacillus; s__ | 4-HPVA | 2.055 | 8.242 | 0.249 | 0.804 | 0.499 |
| k__Bacteria; p__Proteobacteria; c__Gammaproteobacteria; o__Enterobacteriales; f__Enterobacteriaceae; g__; s__ | 4-HPVA | -1.620 | 6.673 | -0.243 | 0.809 | 0.500 |
| k__Bacteria; p__Actinobacteria; c__Coriobacteriia; o__Coriobacteriales; f__Coriobacteriaceae; g__Adlercreutzia; s__ | 4-HPVA | -0.078 | 0.471 | -0.166 | 0.868 | 0.517 |
| k__Bacteria; p__Firmicutes; c__Bacilli; o__Lactobacillales; f__Enterococcaceae; g__; s__ | 4-HPVA | 0.086 | 0.630 | 0.136 | 0.892 | 0.518 |
| k__Bacteria; p__Firmicutes; c__Bacilli; o__Lactobacillales; f__Lactobacillaceae; g__Lactobacillus; s__ | 4-HPVA | 1.005 | 7.578 | 0.133 | 0.895 | 0.518 |
| k__Bacteria; p__Firmicutes; c__Bacilli; o__Lactobacillales; f__Enterococcaceae; g__Enterococcus; s__ | 4-HPVA | -0.297 | 2.657 | -0.112 | 0.911 | 0.522 |
| k__Bacteria; p__Firmicutes; c__Erysipelotrichi; o__Erysipelotrichales; f__Erysipelotrichaceae; g__Allobaculum; s__ | 4-HPVA | 0.047 | 0.684 | 0.068 | 0.946 | 0.531 |
| k__Bacteria; p__Firmicutes; c__Bacilli; o__Lactobacillales; f__Lactobacillaceae; g__Lactobacillus; s__ | 4-HPVA | 0.007 | 0.432 | 0.017 | 0.986 | 0.540 |
| k__Bacteria; p__Firmicutes; c__Bacilli; o__Lactobacillales; f__Aerococcaceae; g__; s__ | 4-HBA | -0.006 | 0.002 | -3.292 | 0.002 | 0.010 |
| k__Bacteria; p__Firmicutes; c__Bacilli; o__Bacillales; f__Planococcaceae; g__Sporosarcina; s__ | 4-HBA | -0.007 | 0.002 | -3.027 | 0.004 | 0.020 |
| k__Bacteria; p__Firmicutes; c__Bacilli; o__Bacillales; f__Planococcaceae; g__Sporosarcina; s__ | 4-HBA | -0.005 | 0.002 | -2.856 | 0.006 | 0.027 |
| k__Bacteria; p__Firmicutes; c__Bacilli; o__Bacillales; f__Planococcaceae; g__Sporosarcina; s__ | 4-HBA | -0.006 | 0.002 | -2.844 | 0.006 | 0.027 |
| k__Bacteria; p__Firmicutes; c__Bacilli; o__Lactobacillales; f__Enterococcaceae; g__; s__ | 4-HBA | -0.008 | 0.005 | -1.692 | 0.095 | 0.192 |
| k__Bacteria; p__Firmicutes; c__Clostridia; o__Clostridiales; f__Lachnospiraceae; g__; s__ | 4-HBA | 0.013 | 0.008 | 1.639 | 0.106 | 0.204 |
| k__Bacteria; p__Bacteroidetes; c__Bacteroidia; o__Bacteroidales; f__S24-7; g__; s__ | 4-HBA | 0.078 | 0.048 | 1.635 | 0.107 | 0.204 |
| k__Bacteria; p__Firmicutes; c__Clostridia; o__Clostridiales; f__Ruminococcaceae; g__; s__ | 4-HBA | 0.003 | 0.002 | 1.552 | 0.125 | 0.234 |
| k__Bacteria; p__Firmicutes; c__Bacilli; o__Lactobacillales; f__Lactobacillaceae; g__Lactobacillus; s__ | 4-HBA | -0.092 | 0.061 | -1.528 | 0.131 | 0.240 |
| k__Bacteria; p__Bacteroidetes; c__Bacteroidia; o__Bacteroidales; f__S24-7; g__; s__ | 4-HBA | 0.003 | 0.002 | 1.415 | 0.162 | 0.279 |
| k__Bacteria; p__Verrucomicrobia; c__Verrucomicrobiae; o__Verrucomicrobiales; f__Verrucomicrobiaceae; g__Akkermansia; s__muciniphila | 4-HBA | 0.056 | 0.042 | 1.334 | 0.187 | 0.310 |
| k__Bacteria; p__Proteobacteria; c__Gammaproteobacteria; o__Alteromonadales; f__Shewanellaceae; g__Shewanella; s__algae | 4-HBA | -0.002 | 0.002 | -1.289 | 0.202 | 0.318 |
| k__Bacteria; p__Firmicutes; c__Clostridia; o__Clostridiales; f__; g__; s__ | 4-HBA | 0.006 | 0.004 | 1.245 | 0.217 | 0.331 |
| k__Bacteria; p__Firmicutes; c__Bacilli; o__Lactobacillales; f__Aerococcaceae; g__Aerococcus; s__ | 4-HBA | 0.010 | 0.008 | 1.214 | 0.229 | 0.335 |
| k__Bacteria; p__Bacteroidetes; c__Bacteroidia; o__Bacteroidales; f__S24-7; g__; s__ | 4-HBA | 0.005 | 0.004 | 1.171 | 0.246 | 0.351 |
| k__Bacteria; p__Firmicutes; c__Bacilli; o__Bacillales; f__Staphylococcaceae; g__Staphylococcus; s__succinus | 4-HBA | -0.017 | 0.015 | -1.169 | 0.247 | 0.351 |
| k__Bacteria; p__Actinobacteria; c__Coriobacteriia; o__Coriobacteriales; f__Coriobacteriaceae; g__Adlercreutzia; s__ | 4-HBA | -0.004 | 0.003 | -1.135 | 0.260 | 0.365 |
| k__Bacteria; p__Firmicutes; c__Erysipelotrichi; o__Erysipelotrichales; f__Erysipelotrichaceae; g__; s__ | 4-HBA | 0.018 | 0.016 | 1.122 | 0.266 | 0.366 |
| k__Bacteria; p__Proteobacteria; c__Gammaproteobacteria; o__Xanthomonadales; f__Xanthomonadaceae; g__Stenotrophomonas; s__ | 4-HBA | -0.001 | 0.001 | -1.058 | 0.294 | 0.388 |
| k__Bacteria; p__Firmicutes; c__Erysipelotrichi; o__Erysipelotrichales; f__Erysipelotrichaceae; g__Allobaculum; s__ | 4-HBA | 0.005 | 0.005 | 0.921 | 0.360 | 0.402 |
| k__Bacteria; p__Firmicutes; c__Clostridia; o__Clostridiales; f__Ruminococcaceae; g__; s__ | 4-HBA | 0.002 | 0.002 | 0.915 | 0.363 | 0.402 |
| k__Bacteria; p__Firmicutes; c__Bacilli; o__Bacillales; f__Staphylococcaceae; g__Jeotgalicoccus; s__psychrophilus | 4-HBA | 0.008 | 0.009 | 0.904 | 0.369 | 0.402 |
| k__Bacteria; p__Firmicutes; c__Bacilli; o__Lactobacillales; f__Lactobacillaceae; g__Lactobacillus; s__ | 4-HBA | -0.002 | 0.002 | -0.880 | 0.382 | 0.402 |
| k__Bacteria; p__Actinobacteria; c__Coriobacteriia; o__Coriobacteriales; f__Coriobacteriaceae; g__Adlercreutzia; s__ | 4-HBA | 0.002 | 0.003 | 0.877 | 0.383 | 0.402 |
| k__Bacteria; p__Proteobacteria; c__Gammaproteobacteria; o__Pseudomonadales; f__Pseudomonadaceae; g__Pseudomonas; s__ | 4-HBA | -0.002 | 0.002 | -0.877 | 0.383 | 0.402 |
| k__Bacteria; p__Actinobacteria; c__Coriobacteriia; o__Coriobacteriales; f__Coriobacteriaceae; g__Adlercreutzia; s__ | 4-HBA | 0.003 | 0.004 | 0.847 | 0.400 | 0.407 |
| k__Bacteria; p__Proteobacteria; c__Gammaproteobacteria; o__Pseudomonadales; f__Pseudomonadaceae; g__Pseudomonas; s__ | 4-HBA | -0.001 | 0.001 | -0.833 | 0.408 | 0.408 |
| k__Bacteria; p__Proteobacteria; c__Gammaproteobacteria; o__Pseudomonadales; f__Pseudomonadaceae; g__Pseudomonas; s__veronii | 4-HBA | -0.001 | 0.001 | -0.810 | 0.421 | 0.409 |
| k__Bacteria; p__Actinobacteria; c__Actinobacteria; o__Bifidobacteriales; f__Bifidobacteriaceae; g__Bifidobacterium; s__ | 4-HBA | -0.002 | 0.003 | -0.806 | 0.423 | 0.409 |
| k__Bacteria; p__Firmicutes; c__Clostridia; o__Clostridiales; f__; g__; s__ | 4-HBA | 0.001 | 0.002 | 0.798 | 0.427 | 0.409 |
| k__Bacteria; p__Actinobacteria; c__Actinobacteria; o__Bifidobacteriales; f__Bifidobacteriaceae; g__Bifidobacterium; s__pseudolongum | 4-HBA | -0.144 | 0.180 | -0.795 | 0.429 | 0.409 |
| k__Bacteria; p__Proteobacteria; c__Gammaproteobacteria; o__Pseudomonadales; f__Pseudomonadaceae; g__Pseudomonas; s__veronii | 4-HBA | -0.091 | 0.115 | -0.793 | 0.430 | 0.409 |
| k__Bacteria; p__Proteobacteria; c__Betaproteobacteria; o__Burkholderiales; f__Alcaligenaceae; g__Achromobacter; s__ | 4-HBA | -0.001 | 0.002 | -0.750 | 0.456 | 0.427 |
| k__Bacteria; p__Firmicutes; c__Clostridia; o__Clostridiales; f__; g__; s__ | 4-HBA | 0.002 | 0.002 | 0.726 | 0.470 | 0.429 |
| k__Bacteria; p__Firmicutes; c__Clostridia; o__Clostridiales; f__; g__; s__ | 4-HBA | 0.008 | 0.012 | 0.647 | 0.520 | 0.445 |
| k__Bacteria; p__Proteobacteria; c__Gammaproteobacteria; o__Xanthomonadales; f__Sinobacteraceae; g__Nevskia; s__ | 4-HBA | -0.002 | 0.003 | -0.634 | 0.528 | 0.446 |
| k__Bacteria; p__Proteobacteria; c__Gammaproteobacteria; o__Enterobacteriales; f__Enterobacteriaceae; g__; s__ | 4-HBA | 0.031 | 0.049 | 0.626 | 0.534 | 0.446 |
| k__Bacteria; p__Bacteroidetes; c__[Saprospirae]; o__[Saprospirales]; f__Chitinophagaceae; g__Sediminibacterium; s__ | 4-HBA | 0.000 | 0.001 | -0.609 | 0.544 | 0.446 |
| k__Bacteria; p__Firmicutes; c__Clostridia; o__Clostridiales; f__Peptostreptococcaceae; g__; s__ | 4-HBA | -0.003 | 0.004 | -0.603 | 0.549 | 0.446 |
| k__Bacteria; p__Bacteroidetes; c__Bacteroidia; o__Bacteroidales; f__S24-7; g__; s__ | 4-HBA | 0.042 | 0.073 | 0.582 | 0.562 | 0.447 |
| k__Bacteria; p__Firmicutes; c__Clostridia; o__Clostridiales; f__; g__; s__ | 4-HBA | 0.002 | 0.004 | 0.580 | 0.564 | 0.447 |
| k__Bacteria; p__Bacteroidetes; c__Bacteroidia; o__Bacteroidales; f__S24-7; g__; s__ | 4-HBA | 0.001 | 0.002 | 0.568 | 0.572 | 0.452 |
| k__Bacteria; p__Proteobacteria; c__Gammaproteobacteria; o__Enterobacteriales; f__Enterobacteriaceae; g__; s__ | 4-HBA | 0.002 | 0.004 | 0.548 | 0.585 | 0.454 |
| k__Bacteria; p__Proteobacteria; c__Alphaproteobacteria; o__Caulobacterales; f__Caulobacteraceae; g__; s__ | 4-HBA | -0.001 | 0.001 | -0.534 | 0.595 | 0.458 |
| k__Bacteria; p__Firmicutes; c__Bacilli; o__Lactobacillales; f__Lactobacillaceae; g__Lactobacillus; s__ | 4-HBA | -0.002 | 0.003 | -0.533 | 0.596 | 0.458 |
| k__Bacteria; p__Firmicutes; c__Bacilli; o__Lactobacillales; f__Lactobacillaceae; g__Lactobacillus; s__ | 4-HBA | -0.029 | 0.056 | -0.514 | 0.609 | 0.462 |
| k__Bacteria; p__Firmicutes; c__Clostridia; o__Clostridiales; f__Lachnospiraceae; g__; s__ | 4-HBA | 0.007 | 0.013 | 0.507 | 0.614 | 0.464 |
| k__Bacteria; p__Proteobacteria; c__Gammaproteobacteria; o__Enterobacteriales; f__Enterobacteriaceae; g__Pantoea; s__agglomerans | 4-HBA | 0.054 | 0.116 | 0.468 | 0.641 | 0.469 |
| k__Bacteria; p__Proteobacteria; c__Gammaproteobacteria; o__Enterobacteriales; f__Enterobacteriaceae; g__; s__ | 4-HBA | 0.002 | 0.004 | 0.468 | 0.641 | 0.469 |
| k__Bacteria; p__Firmicutes; c__Clostridia; o__Clostridiales; f__Clostridiaceae; g__; s__ | 4-HBA | -0.001 | 0.001 | -0.453 | 0.652 | 0.471 |
| k__Bacteria; p__Firmicutes; c__Clostridia; o__Clostridiales; f__Lachnospiraceae; g__Dorea; s__ | 4-HBA | -0.001 | 0.003 | -0.416 | 0.679 | 0.471 |
| k__Bacteria; p__Firmicutes; c__Clostridia; o__Clostridiales; f__Ruminococcaceae; g__; s__ | 4-HBA | 0.002 | 0.006 | 0.384 | 0.702 | 0.474 |
| k__Bacteria; p__Proteobacteria; c__Gammaproteobacteria; o__Oceanospirillales; f__Halomonadaceae; g__Halomonas; s__ | 4-HBA | -0.001 | 0.002 | -0.369 | 0.713 | 0.475 |
| k__Bacteria; p__Actinobacteria; c__Coriobacteriia; o__Coriobacteriales; f__Coriobacteriaceae; g__; s__ | 4-HBA | 0.025 | 0.069 | 0.364 | 0.717 | 0.475 |
| k__Bacteria; p__Firmicutes; c__Bacilli; o__Turicibacterales; f__Turicibacteraceae; g__Turicibacter; s__ | 4-HBA | 0.001 | 0.004 | 0.344 | 0.732 | 0.478 |
| k__Bacteria; p__Firmicutes; c__Bacilli; o__Bacillales; f__Staphylococcaceae; g__Staphylococcus; s__sciuri | 4-HBA | 0.007 | 0.024 | 0.300 | 0.765 | 0.484 |
| k__Bacteria; p__Proteobacteria; c__Alphaproteobacteria; o__Rhizobiales; f__Bradyrhizobiaceae; g__Bradyrhizobium; s__ | 4-HBA | 0.000 | 0.001 | -0.240 | 0.811 | 0.500 |
| k__Bacteria; p__Proteobacteria; c__Gammaproteobacteria; o__Oceanospirillales; f__Halomonadaceae; g__Halomonas; s__ | 4-HBA | 0.000 | 0.002 | -0.223 | 0.824 | 0.504 |
| k__Bacteria; p__Firmicutes; c__Bacilli; o__Lactobacillales; f__Streptococcaceae; g__Streptococcus; s__ | 4-HBA | -0.002 | 0.008 | -0.205 | 0.838 | 0.509 |
| k__Bacteria; p__Firmicutes; c__Clostridia; o__Clostridiales; f__Lachnospiraceae; g__; s__ | 4-HBA | 0.001 | 0.006 | 0.183 | 0.856 | 0.514 |
| k__Bacteria; p__Firmicutes; c__Clostridia; o__Clostridiales; f__; g__; s__ | 4-HBA | -0.001 | 0.004 | -0.160 | 0.874 | 0.518 |
| k__Bacteria; p__Firmicutes; c__Bacilli; o__Lactobacillales; f__Enterococcaceae; g__Enterococcus; s__ | 4-HBA | 0.000 | 0.020 | 0.001 | 0.999 | 0.545 |
| k__Bacteria; p__Firmicutes; c__Clostridia; o__Clostridiales; f__Ruminococcaceae; g__; s__ | 3-HPPA | 0.200 | 0.036 | 5.548 | 0.000 | 0.000 |
| k__Bacteria; p__Bacteroidetes; c__Bacteroidia; o__Bacteroidales; f__S24-7; g__; s__ | 3-HPPA | 0.260 | 0.054 | 4.832 | 0.000 | 0.000 |
| k__Bacteria; p__Firmicutes; c__Clostridia; o__Clostridiales; f__Lachnospiraceae; g__; s__ | 3-HPPA | 0.788 | 0.222 | 3.544 | 0.001 | 0.006 |
| k__Bacteria; p__Firmicutes; c__Erysipelotrichi; o__Erysipelotrichales; f__Erysipelotrichaceae; g__; s__ | 3-HPPA | 0.787 | 0.238 | 3.311 | 0.002 | 0.010 |
| k__Bacteria; p__Verrucomicrobia; c__Verrucomicrobiae; o__Verrucomicrobiales; f__Verrucomicrobiaceae; g__Akkermansia; s__muciniphila | 3-HPPA | 2.716 | 0.866 | 3.136 | 0.003 | 0.015 |
| k__Bacteria; p__Firmicutes; c__Clostridia; o__Clostridiales; f__Lachnospiraceae; g__; s__ | 3-HPPA | 0.088 | 0.030 | 2.940 | 0.005 | 0.023 |
| k__Bacteria; p__Firmicutes; c__Clostridia; o__Clostridiales; f__; g__; s__ | 3-HPPA | 0.327 | 0.113 | 2.898 | 0.005 | 0.025 |
| k__Bacteria; p__Firmicutes; c__Clostridia; o__Clostridiales; f__Ruminococcaceae; g__; s__ | 3-HPPA | 0.161 | 0.056 | 2.895 | 0.005 | 0.025 |
| k__Bacteria; p__Firmicutes; c__Clostridia; o__Clostridiales; f__; g__; s__ | 3-HPPA | 0.104 | 0.038 | 2.746 | 0.008 | 0.034 |
| k__Bacteria; p__Bacteroidetes; c__Bacteroidia; o__Bacteroidales; f__S24-7; g__; s__ | 3-HPPA | 3.645 | 1.333 | 2.733 | 0.008 | 0.035 |
| k__Bacteria; p__Firmicutes; c__Clostridia; o__Clostridiales; f__; g__; s__ | 3-HPPA | 0.217 | 0.085 | 2.563 | 0.013 | 0.048 |
| k__Bacteria; p__Firmicutes; c__Clostridia; o__Clostridiales; f__; g__; s__ | 3-HPPA | 0.134 | 0.053 | 2.524 | 0.014 | 0.051 |
| k__Bacteria; p__Actinobacteria; c__Coriobacteriia; o__Coriobacteriales; f__Coriobacteriaceae; g__Adlercreutzia; s__ | 3-HPPA | 0.125 | 0.075 | 1.665 | 0.101 | 0.199 |
| k__Bacteria; p__Firmicutes; c__Clostridia; o__Clostridiales; f__Lachnospiraceae; g__Dorea; s__ | 3-HPPA | 0.132 | 0.087 | 1.521 | 0.133 | 0.240 |
| k__Bacteria; p__Firmicutes; c__Clostridia; o__Clostridiales; f__Clostridiaceae; g__; s__ | 3-HPPA | -0.072 | 0.057 | -1.268 | 0.209 | 0.325 |
| k__Bacteria; p__Firmicutes; c__Bacilli; o__Lactobacillales; f__Aerococcaceae; g__; s__ | 3-HPPA | -0.066 | 0.060 | -1.103 | 0.274 | 0.374 |
| k__Bacteria; p__Actinobacteria; c__Actinobacteria; o__Bifidobacteriales; f__Bifidobacteriaceae; g__Bifidobacterium; s__ | 3-HPPA | -0.083 | 0.077 | -1.069 | 0.289 | 0.387 |
| k__Bacteria; p__Actinobacteria; c__Actinobacteria; o__Bifidobacteriales; f__Bifidobacteriaceae; g__Bifidobacterium; s__pseudolongum | 3-HPPA | -5.457 | 5.335 | -1.023 | 0.310 | 0.399 |
| k__Bacteria; p__Firmicutes; c__Bacilli; o__Bacillales; f__Planococcaceae; g__Sporosarcina; s__ | 3-HPPA | -0.056 | 0.055 | -1.019 | 0.312 | 0.399 |
| k__Bacteria; p__Firmicutes; c__Bacilli; o__Bacillales; f__Planococcaceae; g__Sporosarcina; s__ | 3-HPPA | -0.074 | 0.073 | -1.014 | 0.314 | 0.399 |
| k__Bacteria; p__Firmicutes; c__Bacilli; o__Lactobacillales; f__Lactobacillaceae; g__Lactobacillus; s__ | 3-HPPA | -0.060 | 0.060 | -1.004 | 0.319 | 0.400 |
| k__Bacteria; p__Firmicutes; c__Bacilli; o__Bacillales; f__Staphylococcaceae; g__Staphylococcus; s__sciuri | 3-HPPA | -0.694 | 0.706 | -0.983 | 0.329 | 0.402 |
| k__Bacteria; p__Firmicutes; c__Clostridia; o__Clostridiales; f__Ruminococcaceae; g__; s__ | 3-HPPA | -0.173 | 0.183 | -0.942 | 0.350 | 0.402 |
| k__Bacteria; p__Firmicutes; c__Bacilli; o__Lactobacillales; f__Lactobacillaceae; g__Lactobacillus; s__ | 3-HPPA | -1.527 | 1.649 | -0.926 | 0.358 | 0.402 |
| k__Bacteria; p__Firmicutes; c__Clostridia; o__Clostridiales; f__; g__; s__ | 3-HPPA | -0.114 | 0.124 | -0.915 | 0.363 | 0.402 |
| k__Bacteria; p__Firmicutes; c__Erysipelotrichi; o__Erysipelotrichales; f__Erysipelotrichaceae; g__Allobaculum; s__ | 3-HPPA | -0.136 | 0.149 | -0.915 | 0.363 | 0.402 |
| k__Bacteria; p__Actinobacteria; c__Coriobacteriia; o__Coriobacteriales; f__Coriobacteriaceae; g__Adlercreutzia; s__ | 3-HPPA | 0.065 | 0.072 | 0.909 | 0.367 | 0.402 |
| k__Bacteria; p__Firmicutes; c__Bacilli; o__Bacillales; f__Planococcaceae; g__Sporosarcina; s__ | 3-HPPA | -0.061 | 0.067 | -0.908 | 0.367 | 0.402 |
| k__Bacteria; p__Firmicutes; c__Bacilli; o__Turicibacterales; f__Turicibacteraceae; g__Turicibacter; s__ | 3-HPPA | -0.108 | 0.120 | -0.898 | 0.373 | 0.402 |
| k__Bacteria; p__Firmicutes; c__Bacilli; o__Lactobacillales; f__Lactobacillaceae; g__Lactobacillus; s__ | 3-HPPA | -0.084 | 0.094 | -0.897 | 0.373 | 0.402 |
| k__Bacteria; p__Actinobacteria; c__Coriobacteriia; o__Coriobacteriales; f__Coriobacteriaceae; g__; s__ | 3-HPPA | -1.803 | 2.023 | -0.891 | 0.376 | 0.402 |
| k__Bacteria; p__Bacteroidetes; c__Bacteroidia; o__Bacteroidales; f__S24-7; g__; s__ | 3-HPPA | 1.745 | 2.095 | 0.833 | 0.408 | 0.408 |
| k__Bacteria; p__Firmicutes; c__Bacilli; o__Bacillales; f__Staphylococcaceae; g__Staphylococcus; s__succinus | 3-HPPA | -0.362 | 0.436 | -0.831 | 0.409 | 0.408 |
| k__Bacteria; p__Bacteroidetes; c__Bacteroidia; o__Bacteroidales; f__S24-7; g__; s__ | 3-HPPA | -0.100 | 0.124 | -0.810 | 0.421 | 0.409 |
| k__Bacteria; p__Bacteroidetes; c__Bacteroidia; o__Bacteroidales; f__S24-7; g__; s__ | 3-HPPA | 0.038 | 0.054 | 0.717 | 0.476 | 0.431 |
| k__Bacteria; p__Proteobacteria; c__Gammaproteobacteria; o__Xanthomonadales; f__Xanthomonadaceae; g__Stenotrophomonas; s__ | 3-HPPA | -0.019 | 0.027 | -0.702 | 0.485 | 0.436 |
| k__Bacteria; p__Proteobacteria; c__Gammaproteobacteria; o__Alteromonadales; f__Shewanellaceae; g__Shewanella; s__algae | 3-HPPA | -0.036 | 0.055 | -0.650 | 0.518 | 0.445 |
| k__Bacteria; p__Firmicutes; c__Clostridia; o__Clostridiales; f__Lachnospiraceae; g__; s__ | 3-HPPA | 0.020 | 0.032 | 0.632 | 0.530 | 0.446 |
| k__Bacteria; p__Firmicutes; c__Bacilli; o__Lactobacillales; f__Aerococcaceae; g__Aerococcus; s__ | 3-HPPA | -0.150 | 0.248 | -0.602 | 0.549 | 0.446 |
| k__Bacteria; p__Proteobacteria; c__Gammaproteobacteria; o__Oceanospirillales; f__Halomonadaceae; g__Halomonas; s__ | 3-HPPA | 0.037 | 0.069 | 0.528 | 0.599 | 0.459 |
| k__Bacteria; p__Proteobacteria; c__Gammaproteobacteria; o__Oceanospirillales; f__Halomonadaceae; g__Halomonas; s__ | 3-HPPA | 0.024 | 0.050 | 0.492 | 0.625 | 0.465 |
| k__Bacteria; p__Firmicutes; c__Bacilli; o__Lactobacillales; f__Lactobacillaceae; g__Lactobacillus; s__ | 3-HPPA | -0.858 | 1.746 | -0.492 | 0.625 | 0.465 |
| k__Bacteria; p__Firmicutes; c__Bacilli; o__Bacillales; f__Staphylococcaceae; g__Jeotgalicoccus; s__psychrophilus | 3-HPPA | -0.124 | 0.264 | -0.470 | 0.640 | 0.469 |
| k__Bacteria; p__Firmicutes; c__Bacilli; o__Lactobacillales; f__Enterococcaceae; g__; s__ | 3-HPPA | -0.063 | 0.138 | -0.457 | 0.649 | 0.471 |
| k__Bacteria; p__Firmicutes; c__Bacilli; o__Lactobacillales; f__Streptococcaceae; g__Streptococcus; s__ | 3-HPPA | -0.091 | 0.232 | -0.394 | 0.695 | 0.471 |
| k__Bacteria; p__Actinobacteria; c__Coriobacteriia; o__Coriobacteriales; f__Coriobacteriaceae; g__Adlercreutzia; s__ | 3-HPPA | -0.033 | 0.103 | -0.320 | 0.750 | 0.483 |
| k__Bacteria; p__Firmicutes; c__Clostridia; o__Clostridiales; f__; g__; s__ | 3-HPPA | -0.038 | 0.118 | -0.318 | 0.751 | 0.483 |
| k__Bacteria; p__Proteobacteria; c__Gammaproteobacteria; o__Enterobacteriales; f__Enterobacteriaceae; g__; s__ | 3-HPPA | -0.455 | 1.462 | -0.311 | 0.757 | 0.483 |
| k__Bacteria; p__Proteobacteria; c__Gammaproteobacteria; o__Pseudomonadales; f__Pseudomonadaceae; g__Pseudomonas; s__veronii | 3-HPPA | 0.005 | 0.029 | 0.188 | 0.852 | 0.512 |
| k__Bacteria; p__Proteobacteria; c__Alphaproteobacteria; o__Caulobacterales; f__Caulobacteraceae; g__; s__ | 3-HPPA | 0.006 | 0.032 | 0.176 | 0.861 | 0.515 |
| k__Bacteria; p__Proteobacteria; c__Gammaproteobacteria; o__Enterobacteriales; f__Enterobacteriaceae; g__; s__ | 3-HPPA | -0.017 | 0.116 | -0.149 | 0.882 | 0.518 |
| k__Bacteria; p__Firmicutes; c__Clostridia; o__Clostridiales; f__Peptostreptococcaceae; g__; s__ | 3-HPPA | 0.020 | 0.131 | 0.149 | 0.882 | 0.518 |
| k__Bacteria; p__Proteobacteria; c__Gammaproteobacteria; o__Enterobacteriales; f__Enterobacteriaceae; g__Pantoea; s__agglomerans | 3-HPPA | -0.505 | 3.436 | -0.147 | 0.884 | 0.518 |
| k__Bacteria; p__Firmicutes; c__Bacilli; o__Lactobacillales; f__Enterococcaceae; g__Enterococcus; s__ | 3-HPPA | -0.083 | 0.582 | -0.142 | 0.887 | 0.518 |
| k__Bacteria; p__Bacteroidetes; c__[Saprospirae]; o__[Saprospirales]; f__Chitinophagaceae; g__Sediminibacterium; s__ | 3-HPPA | -0.002 | 0.016 | -0.136 | 0.892 | 0.518 |
| k__Bacteria; p__Proteobacteria; c__Gammaproteobacteria; o__Pseudomonadales; f__Pseudomonadaceae; g__Pseudomonas; s__ | 3-HPPA | 0.004 | 0.039 | 0.097 | 0.923 | 0.526 |
| k__Bacteria; p__Proteobacteria; c__Gammaproteobacteria; o__Enterobacteriales; f__Enterobacteriaceae; g__; s__ | 3-HPPA | -0.010 | 0.121 | -0.082 | 0.935 | 0.529 |
| k__Bacteria; p__Proteobacteria; c__Alphaproteobacteria; o__Rhizobiales; f__Bradyrhizobiaceae; g__Bradyrhizobium; s__ | 3-HPPA | 0.002 | 0.023 | 0.077 | 0.939 | 0.530 |
| k__Bacteria; p__Proteobacteria; c__Gammaproteobacteria; o__Pseudomonadales; f__Pseudomonadaceae; g__Pseudomonas; s__veronii | 3-HPPA | 0.232 | 3.419 | 0.068 | 0.946 | 0.531 |
| k__Bacteria; p__Proteobacteria; c__Gammaproteobacteria; o__Pseudomonadales; f__Pseudomonadaceae; g__Pseudomonas; s__ | 3-HPPA | 0.004 | 0.075 | 0.048 | 0.962 | 0.536 |
| k__Bacteria; p__Proteobacteria; c__Betaproteobacteria; o__Burkholderiales; f__Alcaligenaceae; g__Achromobacter; s__ | 3-HPPA | -0.001 | 0.050 | -0.023 | 0.982 | 0.540 |
| k__Bacteria; p__Proteobacteria; c__Gammaproteobacteria; o__Xanthomonadales; f__Sinobacteraceae; g__Nevskia; s__ | 3-HPPA | 0.001 | 0.091 | 0.016 | 0.987 | 0.540 |
| k__Bacteria; p__Firmicutes; c__Bacilli; o__Bacillales; f__Staphylococcaceae; g__Staphylococcus; s__sciuri | 3,4-diHPAA | 0.878 | 0.294 | 2.982 | 0.004 | 0.023 |
| k__Bacteria; p__Actinobacteria; c__Coriobacteriia; o__Coriobacteriales; f__Coriobacteriaceae; g__; s__ | 3,4-diHPAA | 4.999 | 1.926 | 2.595 | 0.012 | 0.046 |
| k__Bacteria; p__Firmicutes; c__Bacilli; o__Bacillales; f__Planococcaceae; g__Sporosarcina; s__ | 3,4-diHPAA | 0.167 | 0.082 | 2.046 | 0.046 | 0.116 |
| k__Bacteria; p__Firmicutes; c__Bacilli; o__Lactobacillales; f__Aerococcaceae; g__; s__ | 3,4-diHPAA | 0.132 | 0.067 | 1.971 | 0.054 | 0.128 |
| k__Bacteria; p__Firmicutes; c__Bacilli; o__Lactobacillales; f__Aerococcaceae; g__Aerococcus; s__ | 3,4-diHPAA | 0.324 | 0.186 | 1.742 | 0.087 | 0.182 |
| k__Bacteria; p__Firmicutes; c__Bacilli; o__Bacillales; f__Planococcaceae; g__Sporosarcina; s__ | 3,4-diHPAA | 0.107 | 0.062 | 1.730 | 0.090 | 0.184 |
| k__Bacteria; p__Firmicutes; c__Bacilli; o__Bacillales; f__Planococcaceae; g__Sporosarcina; s__ | 3,4-diHPAA | 0.126 | 0.076 | 1.659 | 0.103 | 0.201 |
| k__Bacteria; p__Proteobacteria; c__Gammaproteobacteria; o__Enterobacteriales; f__Enterobacteriaceae; g__; s__ | 3,4-diHPAA | -2.468 | 1.657 | -1.490 | 0.142 | 0.250 |
| k__Bacteria; p__Bacteroidetes; c__Bacteroidia; o__Bacteroidales; f__S24-7; g__; s__ | 3,4-diHPAA | -0.096 | 0.074 | -1.302 | 0.199 | 0.318 |
| k__Bacteria; p__Bacteroidetes; c__Bacteroidia; o__Bacteroidales; f__S24-7; g__; s__ | 3,4-diHPAA | -0.042 | 0.037 | -1.142 | 0.259 | 0.364 |
| k__Bacteria; p__Firmicutes; c__Clostridia; o__Clostridiales; f__Lachnospiraceae; g__Dorea; s__ | 3,4-diHPAA | 0.113 | 0.101 | 1.121 | 0.267 | 0.366 |
| k__Bacteria; p__Proteobacteria; c__Gammaproteobacteria; o__Oceanospirillales; f__Halomonadaceae; g__Halomonas; s__ | 3,4-diHPAA | -0.050 | 0.050 | -1.017 | 0.314 | 0.399 |
| k__Bacteria; p__Actinobacteria; c__Coriobacteriia; o__Coriobacteriales; f__Coriobacteriaceae; g__Adlercreutzia; s__ | 3,4-diHPAA | 0.124 | 0.130 | 0.958 | 0.342 | 0.402 |
| k__Bacteria; p__Proteobacteria; c__Gammaproteobacteria; o__Xanthomonadales; f__Sinobacteraceae; g__Nevskia; s__ | 3,4-diHPAA | -0.086 | 0.090 | -0.956 | 0.343 | 0.402 |
| k__Bacteria; p__Proteobacteria; c__Gammaproteobacteria; o__Enterobacteriales; f__Enterobacteriaceae; g__Pantoea; s__agglomerans | 3,4-diHPAA | 3.684 | 3.939 | 0.935 | 0.354 | 0.402 |
| k__Bacteria; p__Proteobacteria; c__Gammaproteobacteria; o__Enterobacteriales; f__Enterobacteriaceae; g__; s__ | 3,4-diHPAA | 0.125 | 0.133 | 0.935 | 0.354 | 0.402 |
| k__Bacteria; p__Proteobacteria; c__Gammaproteobacteria; o__Pseudomonadales; f__Pseudomonadaceae; g__Pseudomonas; s__veronii | 3,4-diHPAA | -0.027 | 0.029 | -0.934 | 0.354 | 0.402 |
| k__Bacteria; p__Firmicutes; c__Bacilli; o__Turicibacterales; f__Turicibacteraceae; g__Turicibacter; s__ | 3,4-diHPAA | -0.125 | 0.138 | -0.905 | 0.370 | 0.402 |
| k__Bacteria; p__Proteobacteria; c__Gammaproteobacteria; o__Oceanospirillales; f__Halomonadaceae; g__Halomonas; s__ | 3,4-diHPAA | -0.061 | 0.069 | -0.885 | 0.380 | 0.402 |
| k__Bacteria; p__Actinobacteria; c__Coriobacteriia; o__Coriobacteriales; f__Coriobacteriaceae; g__Adlercreutzia; s__ | 3,4-diHPAA | 0.070 | 0.084 | 0.826 | 0.413 | 0.409 |
| k__Bacteria; p__Proteobacteria; c__Gammaproteobacteria; o__Pseudomonadales; f__Pseudomonadaceae; g__Pseudomonas; s__ | 3,4-diHPAA | -0.061 | 0.075 | -0.822 | 0.415 | 0.409 |
| k__Bacteria; p__Firmicutes; c__Bacilli; o__Bacillales; f__Staphylococcaceae; g__Staphylococcus; s__succinus | 3,4-diHPAA | 0.396 | 0.492 | 0.805 | 0.424 | 0.409 |
| k__Bacteria; p__Proteobacteria; c__Gammaproteobacteria; o__Enterobacteriales; f__Enterobacteriaceae; g__; s__ | 3,4-diHPAA | 0.109 | 0.139 | 0.783 | 0.437 | 0.414 |
| k__Bacteria; p__Proteobacteria; c__Gammaproteobacteria; o__Pseudomonadales; f__Pseudomonadaceae; g__Pseudomonas; s__veronii | 3,4-diHPAA | -2.507 | 3.453 | -0.726 | 0.471 | 0.429 |
| k__Bacteria; p__Actinobacteria; c__Actinobacteria; o__Bifidobacteriales; f__Bifidobacteriaceae; g__Bifidobacterium; s__ | 3,4-diHPAA | 0.058 | 0.084 | 0.690 | 0.493 | 0.439 |
| k__Bacteria; p__Firmicutes; c__Bacilli; o__Lactobacillales; f__Lactobacillaceae; g__Lactobacillus; s__ | 3,4-diHPAA | 0.071 | 0.105 | 0.679 | 0.500 | 0.442 |
| k__Bacteria; p__Firmicutes; c__Bacilli; o__Bacillales; f__Staphylococcaceae; g__Jeotgalicoccus; s__psychrophilus | 3,4-diHPAA | 0.035 | 0.052 | 0.677 | 0.501 | 0.442 |
| k__Bacteria; p__Bacteroidetes; c__Bacteroidia; o__Bacteroidales; f__S24-7; g__; s__ | 3,4-diHPAA | -0.021 | 0.032 | -0.653 | 0.517 | 0.445 |
| k__Bacteria; p__Firmicutes; c__Bacilli; o__Lactobacillales; f__Lactobacillaceae; g__Lactobacillus; s__ | 3,4-diHPAA | 1.167 | 1.825 | 0.640 | 0.525 | 0.446 |
| k__Bacteria; p__Actinobacteria; c__Actinobacteria; o__Bifidobacteriales; f__Bifidobacteriaceae; g__Bifidobacterium; s__pseudolongum | 3,4-diHPAA | 3.567 | 5.805 | 0.615 | 0.542 | 0.446 |
| k__Bacteria; p__Proteobacteria; c__Betaproteobacteria; o__Burkholderiales; f__Alcaligenaceae; g__Achromobacter; s__ | 3,4-diHPAA | -0.028 | 0.051 | -0.557 | 0.580 | 0.454 |
| k__Bacteria; p__Firmicutes; c__Clostridia; o__Clostridiales; f__; g__; s__ | 3,4-diHPAA | -0.068 | 0.130 | -0.523 | 0.603 | 0.460 |
| k__Bacteria; p__Proteobacteria; c__Gammaproteobacteria; o__Pseudomonadales; f__Pseudomonadaceae; g__Pseudomonas; s__ | 3,4-diHPAA | -0.020 | 0.039 | -0.497 | 0.621 | 0.465 |
| k__Bacteria; p__Firmicutes; c__Clostridia; o__Clostridiales; f__Ruminococcaceae; g__; s__ | 3,4-diHPAA | 0.017 | 0.034 | 0.491 | 0.626 | 0.465 |
| k__Bacteria; p__Firmicutes; c__Erysipelotrichi; o__Erysipelotrichales; f__Erysipelotrichaceae; g__; s__ | 3,4-diHPAA | 0.239 | 0.503 | 0.474 | 0.638 | 0.469 |
| k__Bacteria; p__Firmicutes; c__Clostridia; o__Clostridiales; f__; g__; s__ | 3,4-diHPAA | 0.185 | 0.399 | 0.463 | 0.645 | 0.471 |
| k__Bacteria; p__Firmicutes; c__Clostridia; o__Clostridiales; f__; g__; s__ | 3,4-diHPAA | 0.024 | 0.054 | 0.445 | 0.658 | 0.471 |
| k__Bacteria; p__Proteobacteria; c__Gammaproteobacteria; o__Xanthomonadales; f__Xanthomonadaceae; g__Stenotrophomonas; s__ | 3,4-diHPAA | -0.012 | 0.028 | -0.438 | 0.663 | 0.471 |
| k__Bacteria; p__Firmicutes; c__Clostridia; o__Clostridiales; f__; g__; s__ | 3,4-diHPAA | 0.049 | 0.115 | 0.430 | 0.669 | 0.471 |
| k__Bacteria; p__Firmicutes; c__Bacilli; o__Lactobacillales; f__Lactobacillaceae; g__Lactobacillus; s__ | 3,4-diHPAA | 0.028 | 0.068 | 0.415 | 0.680 | 0.471 |
| k__Bacteria; p__Bacteroidetes; c__Bacteroidia; o__Bacteroidales; f__S24-7; g__; s__ | 3,4-diHPAA | -0.713 | 1.736 | -0.411 | 0.683 | 0.471 |
| k__Bacteria; p__Firmicutes; c__Bacilli; o__Lactobacillales; f__Streptococcaceae; g__Streptococcus; s__ | 3,4-diHPAA | 0.106 | 0.259 | 0.408 | 0.685 | 0.471 |
| k__Bacteria; p__Verrucomicrobia; c__Verrucomicrobiae; o__Verrucomicrobiales; f__Verrucomicrobiaceae; g__Akkermansia; s__muciniphila | 3,4-diHPAA | 0.446 | 1.118 | 0.399 | 0.692 | 0.471 |
| k__Bacteria; p__Proteobacteria; c__Alphaproteobacteria; o__Caulobacterales; f__Caulobacteraceae; g__; s__ | 3,4-diHPAA | -0.013 | 0.035 | -0.386 | 0.701 | 0.474 |
| k__Bacteria; p__Firmicutes; c__Erysipelotrichi; o__Erysipelotrichales; f__Erysipelotrichaceae; g__Allobaculum; s__ | 3,4-diHPAA | -0.011 | 0.030 | -0.378 | 0.707 | 0.474 |
| k__Bacteria; p__Firmicutes; c__Clostridia; o__Clostridiales; f__; g__; s__ | 3,4-diHPAA | 0.026 | 0.072 | 0.368 | 0.714 | 0.475 |
| k__Bacteria; p__Proteobacteria; c__Alphaproteobacteria; o__Rhizobiales; f__Bradyrhizobiaceae; g__Bradyrhizobium; s__ | 3,4-diHPAA | -0.008 | 0.022 | -0.352 | 0.727 | 0.478 |
| k__Bacteria; p__Firmicutes; c__Clostridia; o__Clostridiales; f__Lachnospiraceae; g__; s__ | 3,4-diHPAA | 0.065 | 0.187 | 0.346 | 0.731 | 0.478 |
| k__Bacteria; p__Firmicutes; c__Clostridia; o__Clostridiales; f__Lachnospiraceae; g__; s__ | 3,4-diHPAA | 0.032 | 0.094 | 0.344 | 0.732 | 0.478 |
| k__Bacteria; p__Firmicutes; c__Clostridia; o__Clostridiales; f__Lachnospiraceae; g__; s__ | 3,4-diHPAA | 0.154 | 0.453 | 0.341 | 0.735 | 0.479 |
| k__Bacteria; p__Bacteroidetes; c__[Saprospirae]; o__[Saprospirales]; f__Chitinophagaceae; g__Sediminibacterium; s__ | 3,4-diHPAA | -0.005 | 0.016 | -0.308 | 0.760 | 0.483 |
| k__Bacteria; p__Firmicutes; c__Clostridia; o__Clostridiales; f__Clostridiaceae; g__; s__ | 3,4-diHPAA | 0.010 | 0.042 | 0.252 | 0.802 | 0.499 |
| k__Bacteria; p__Firmicutes; c__Clostridia; o__Clostridiales; f__Ruminococcaceae; g__; s__ | 3,4-diHPAA | -0.043 | 0.175 | -0.245 | 0.807 | 0.500 |
| k__Bacteria; p__Firmicutes; c__Bacilli; o__Lactobacillales; f__Lactobacillaceae; g__Lactobacillus; s__ | 3,4-diHPAA | -0.408 | 1.975 | -0.207 | 0.837 | 0.509 |
| k__Bacteria; p__Proteobacteria; c__Gammaproteobacteria; o__Alteromonadales; f__Shewanellaceae; g__Shewanella; s__algae | 3,4-diHPAA | -0.012 | 0.058 | -0.201 | 0.842 | 0.510 |
| k__Bacteria; p__Bacteroidetes; c__Bacteroidia; o__Bacteroidales; f__S24-7; g__; s__ | 3,4-diHPAA | -0.110 | 0.632 | -0.175 | 0.862 | 0.515 |
| k__Bacteria; p__Firmicutes; c__Clostridia; o__Clostridiales; f__Peptostreptococcaceae; g__; s__ | 3,4-diHPAA | 0.020 | 0.151 | 0.135 | 0.893 | 0.518 |
| k__Bacteria; p__Firmicutes; c__Bacilli; o__Lactobacillales; f__Enterococcaceae; g__Enterococcus; s__ | 3,4-diHPAA | 0.076 | 0.660 | 0.115 | 0.909 | 0.522 |
| k__Bacteria; p__Firmicutes; c__Bacilli; o__Lactobacillales; f__Enterococcaceae; g__; s__ | 3,4-diHPAA | 0.016 | 0.160 | 0.099 | 0.921 | 0.526 |
| k__Bacteria; p__Actinobacteria; c__Coriobacteriia; o__Coriobacteriales; f__Coriobacteriaceae; g__Adlercreutzia; s__ | 3,4-diHPAA | 0.004 | 0.119 | 0.037 | 0.970 | 0.538 |
| k__Bacteria; p__Firmicutes; c__Clostridia; o__Clostridiales; f__Ruminococcaceae; g__; s__ | 3,4-diHPAA | -0.002 | 0.055 | -0.037 | 0.971 | 0.538 |
| k__Bacteria; p__Firmicutes; c__Clostridia; o__Clostridiales; f__; g__; s__ | 3,4-diHPAA | 0.003 | 0.133 | 0.026 | 0.979 | 0.540 |
| k__Bacteria; p__Proteobacteria; c__Gammaproteobacteria; o__Oceanospirillales; f__Halomonadaceae; g__Halomonas; s__ | (+)-C | 0.394 | 0.179 | 2.198 | 0.031 | 0.088 |
| k__Bacteria; p__Proteobacteria; c__Alphaproteobacteria; o__Caulobacterales; f__Caulobacteraceae; g__; s__ | (+)-C | 0.252 | 0.116 | 2.183 | 0.033 | 0.090 |
| k__Bacteria; p__Proteobacteria; c__Gammaproteobacteria; o__Pseudomonadales; f__Pseudomonadaceae; g__Pseudomonas; s__ | (+)-C | 0.294 | 0.139 | 2.112 | 0.038 | 0.103 |
| k__Bacteria; p__Proteobacteria; c__Gammaproteobacteria; o__Oceanospirillales; f__Halomonadaceae; g__Halomonas; s__ | (+)-C | 0.498 | 0.247 | 2.015 | 0.048 | 0.118 |
| k__Bacteria; p__Proteobacteria; c__Gammaproteobacteria; o__Pseudomonadales; f__Pseudomonadaceae; g__Pseudomonas; s__ | (+)-C | 0.513 | 0.266 | 1.928 | 0.058 | 0.134 |
| k__Bacteria; p__Proteobacteria; c__Gammaproteobacteria; o__Pseudomonadales; f__Pseudomonadaceae; g__Pseudomonas; s__veronii | (+)-C | 0.199 | 0.105 | 1.900 | 0.062 | 0.136 |
| k__Bacteria; p__Proteobacteria; c__Gammaproteobacteria; o__Pseudomonadales; f__Pseudomonadaceae; g__Pseudomonas; s__veronii | (+)-C | 21.793 | 12.289 | 1.773 | 0.081 | 0.173 |
| k__Bacteria; p__Proteobacteria; c__Alphaproteobacteria; o__Rhizobiales; f__Bradyrhizobiaceae; g__Bradyrhizobium; s__ | (+)-C | 0.141 | 0.084 | 1.679 | 0.098 | 0.195 |
| k__Bacteria; p__Proteobacteria; c__Betaproteobacteria; o__Burkholderiales; f__Alcaligenaceae; g__Achromobacter; s__ | (+)-C | 0.273 | 0.178 | 1.532 | 0.130 | 0.240 |
| k__Bacteria; p__Proteobacteria; c__Gammaproteobacteria; o__Xanthomonadales; f__Sinobacteraceae; g__Nevskia; s__ | (+)-C | 0.457 | 0.330 | 1.386 | 0.170 | 0.292 |
| k__Bacteria; p__Firmicutes; c__Bacilli; o__Lactobacillales; f__Streptococcaceae; g__Streptococcus; s__ | (+)-C | -1.125 | 0.844 | -1.333 | 0.187 | 0.310 |
| k__Bacteria; p__Bacteroidetes; c__[Saprospirae]; o__[Saprospirales]; f__Chitinophagaceae; g__Sediminibacterium; s__ | (+)-C | 0.071 | 0.058 | 1.219 | 0.227 | 0.334 |
| k__Bacteria; p__Actinobacteria; c__Actinobacteria; o__Bifidobacteriales; f__Bifidobacteriaceae; g__Bifidobacterium; s__pseudolongum | (+)-C | -23.617 | 19.623 | -1.204 | 0.233 | 0.338 |
| k__Bacteria; p__Actinobacteria; c__Actinobacteria; o__Bifidobacteriales; f__Bifidobacteriaceae; g__Bifidobacterium; s__ | (+)-C | -0.342 | 0.284 | -1.203 | 0.233 | 0.338 |
| k__Bacteria; p__Firmicutes; c__Bacilli; o__Lactobacillales; f__Lactobacillaceae; g__Lactobacillus; s__ | (+)-C | -7.086 | 6.057 | -1.170 | 0.246 | 0.351 |
| k__Bacteria; p__Firmicutes; c__Bacilli; o__Lactobacillales; f__Lactobacillaceae; g__Lactobacillus; s__ | (+)-C | -0.388 | 0.345 | -1.124 | 0.265 | 0.366 |
| k__Bacteria; p__Firmicutes; c__Clostridia; o__Clostridiales; f__Ruminococcaceae; g__; s__ | (+)-C | -0.189 | 0.174 | -1.091 | 0.279 | 0.379 |
| k__Bacteria; p__Firmicutes; c__Bacilli; o__Lactobacillales; f__Aerococcaceae; g__; s__ | (+)-C | -0.235 | 0.222 | -1.059 | 0.293 | 0.388 |
| k__Bacteria; p__Bacteroidetes; c__Bacteroidia; o__Bacteroidales; f__S24-7; g__; s__ | (+)-C | -8.173 | 7.734 | -1.057 | 0.294 | 0.388 |
| k__Bacteria; p__Firmicutes; c__Bacilli; o__Lactobacillales; f__Lactobacillaceae; g__Lactobacillus; s__ | (+)-C | -0.229 | 0.219 | -1.044 | 0.300 | 0.392 |
| k__Bacteria; p__Firmicutes; c__Bacilli; o__Bacillales; f__Planococcaceae; g__Sporosarcina; s__ | (+)-C | -0.274 | 0.270 | -1.015 | 0.314 | 0.399 |
| k__Bacteria; p__Firmicutes; c__Clostridia; o__Clostridiales; f__Ruminococcaceae; g__; s__ | (+)-C | -0.661 | 0.658 | -1.003 | 0.319 | 0.400 |
| k__Bacteria; p__Firmicutes; c__Bacilli; o__Bacillales; f__Planococcaceae; g__Sporosarcina; s__ | (+)-C | -0.203 | 0.203 | -1.003 | 0.319 | 0.400 |
| k__Bacteria; p__Bacteroidetes; c__Bacteroidia; o__Bacteroidales; f__S24-7; g__; s__ | (+)-C | -0.192 | 0.205 | -0.938 | 0.352 | 0.402 |
| k__Bacteria; p__Verrucomicrobia; c__Verrucomicrobiae; o__Verrucomicrobiales; f__Verrucomicrobiaceae; g__Akkermansia; s__muciniphila | (+)-C | -4.273 | 4.586 | -0.932 | 0.355 | 0.402 |
| k__Bacteria; p__Proteobacteria; c__Gammaproteobacteria; o__Alteromonadales; f__Shewanellaceae; g__Shewanella; s__algae | (+)-C | 0.187 | 0.203 | 0.921 | 0.360 | 0.402 |
| k__Bacteria; p__Firmicutes; c__Clostridia; o__Clostridiales; f__; g__; s__ | (+)-C | -0.185 | 0.203 | -0.911 | 0.365 | 0.402 |
| k__Bacteria; p__Actinobacteria; c__Coriobacteriia; o__Coriobacteriales; f__Coriobacteriaceae; g__; s__ | (+)-C | -6.624 | 7.395 | -0.896 | 0.374 | 0.402 |
| k__Bacteria; p__Actinobacteria; c__Coriobacteriia; o__Coriobacteriales; f__Coriobacteriaceae; g__Adlercreutzia; s__ | (+)-C | -0.266 | 0.300 | -0.885 | 0.379 | 0.402 |
| k__Bacteria; p__Firmicutes; c__Bacilli; o__Bacillales; f__Planococcaceae; g__Sporosarcina; s__ | (+)-C | -0.219 | 0.248 | -0.880 | 0.382 | 0.402 |
| k__Bacteria; p__Bacteroidetes; c__Bacteroidia; o__Bacteroidales; f__S24-7; g__; s__ | (+)-C | -4.668 | 5.310 | -0.879 | 0.382 | 0.402 |
| k__Bacteria; p__Firmicutes; c__Bacilli; o__Lactobacillales; f__Aerococcaceae; g__Aerococcus; s__ | (+)-C | -0.790 | 0.912 | -0.866 | 0.390 | 0.404 |
| k__Bacteria; p__Firmicutes; c__Clostridia; o__Clostridiales; f__; g__; s__ | (+)-C | -0.324 | 0.377 | -0.859 | 0.393 | 0.404 |
| k__Bacteria; p__Firmicutes; c__Clostridia; o__Clostridiales; f__; g__; s__ | (+)-C | -0.136 | 0.161 | -0.843 | 0.402 | 0.408 |
| k__Bacteria; p__Firmicutes; c__Bacilli; o__Lactobacillales; f__Lactobacillaceae; g__Lactobacillus; s__ | (+)-C | -5.629 | 6.730 | -0.836 | 0.406 | 0.408 |
| k__Bacteria; p__Firmicutes; c__Clostridia; o__Clostridiales; f__; g__; s__ | (+)-C | -0.197 | 0.236 | -0.836 | 0.406 | 0.408 |
| k__Bacteria; p__Firmicutes; c__Clostridia; o__Clostridiales; f__Clostridiaceae; g__; s__ | (+)-C | -0.160 | 0.195 | -0.821 | 0.415 | 0.409 |
| k__Bacteria; p__Firmicutes; c__Bacilli; o__Bacillales; f__Staphylococcaceae; g__Staphylococcus; s__sciuri | (+)-C | -2.111 | 2.576 | -0.819 | 0.416 | 0.409 |
| k__Bacteria; p__Firmicutes; c__Erysipelotrichi; o__Erysipelotrichales; f__Erysipelotrichaceae; g__; s__ | (+)-C | -1.471 | 1.802 | -0.816 | 0.417 | 0.409 |
| k__Bacteria; p__Actinobacteria; c__Coriobacteriia; o__Coriobacteriales; f__Coriobacteriaceae; g__Adlercreutzia; s__ | (+)-C | -0.347 | 0.436 | -0.794 | 0.430 | 0.409 |
| k__Bacteria; p__Firmicutes; c__Bacilli; o__Bacillales; f__Staphylococcaceae; g__Staphylococcus; s__succinus | (+)-C | -1.230 | 1.606 | -0.766 | 0.446 | 0.422 |
| k__Bacteria; p__Firmicutes; c__Clostridia; o__Clostridiales; f__Ruminococcaceae; g__; s__ | (+)-C | -0.136 | 0.184 | -0.738 | 0.463 | 0.427 |
| k__Bacteria; p__Actinobacteria; c__Coriobacteriia; o__Coriobacteriales; f__Coriobacteriaceae; g__Adlercreutzia; s__ | (+)-C | -0.273 | 0.379 | -0.721 | 0.473 | 0.430 |
| k__Bacteria; p__Firmicutes; c__Clostridia; o__Clostridiales; f__Lachnospiraceae; g__; s__ | (+)-C | -0.620 | 0.888 | -0.699 | 0.487 | 0.436 |
| k__Bacteria; p__Firmicutes; c__Bacilli; o__Lactobacillales; f__Enterococcaceae; g__Enterococcus; s__ | (+)-C | -1.405 | 2.136 | -0.658 | 0.513 | 0.445 |
| k__Bacteria; p__Firmicutes; c__Bacilli; o__Lactobacillales; f__Enterococcaceae; g__; s__ | (+)-C | -0.329 | 0.507 | -0.649 | 0.519 | 0.445 |
| k__Bacteria; p__Firmicutes; c__Bacilli; o__Bacillales; f__Staphylococcaceae; g__Jeotgalicoccus; s__psychrophilus | (+)-C | -0.603 | 0.970 | -0.622 | 0.536 | 0.446 |
| k__Bacteria; p__Firmicutes; c__Clostridia; o__Clostridiales; f__Lachnospiraceae; g__Dorea; s__ | (+)-C | -0.198 | 0.330 | -0.599 | 0.551 | 0.446 |
| k__Bacteria; p__Firmicutes; c__Clostridia; o__Clostridiales; f__; g__; s__ | (+)-C | -0.286 | 0.487 | -0.587 | 0.559 | 0.446 |
| k__Bacteria; p__Firmicutes; c__Bacilli; o__Turicibacterales; f__Turicibacteraceae; g__Turicibacter; s__ | (+)-C | -0.241 | 0.443 | -0.544 | 0.589 | 0.456 |
| k__Bacteria; p__Bacteroidetes; c__Bacteroidia; o__Bacteroidales; f__S24-7; g__; s__ | (+)-C | -0.140 | 0.261 | -0.537 | 0.593 | 0.458 |
| k__Bacteria; p__Firmicutes; c__Clostridia; o__Clostridiales; f__; g__; s__ | (+)-C | -0.685 | 1.318 | -0.520 | 0.605 | 0.460 |
| k__Bacteria; p__Bacteroidetes; c__Bacteroidia; o__Bacteroidales; f__S24-7; g__; s__ | (+)-C | -0.223 | 0.458 | -0.488 | 0.627 | 0.465 |
| k__Bacteria; p__Firmicutes; c__Clostridia; o__Clostridiales; f__Lachnospiraceae; g__; s__ | (+)-C | -0.285 | 0.595 | -0.479 | 0.634 | 0.468 |
| k__Bacteria; p__Firmicutes; c__Clostridia; o__Clostridiales; f__Peptostreptococcaceae; g__; s__ | (+)-C | -0.196 | 0.437 | -0.448 | 0.656 | 0.471 |
| k__Bacteria; p__Firmicutes; c__Clostridia; o__Clostridiales; f__Lachnospiraceae; g__; s__ | (+)-C | -0.645 | 1.443 | -0.447 | 0.656 | 0.471 |
| k__Bacteria; p__Firmicutes; c__Erysipelotrichi; o__Erysipelotrichales; f__Erysipelotrichaceae; g__Allobaculum; s__ | (+)-C | -0.244 | 0.552 | -0.443 | 0.659 | 0.471 |
| k__Bacteria; p__Proteobacteria; c__Gammaproteobacteria; o__Xanthomonadales; f__Xanthomonadaceae; g__Stenotrophomonas; s__ | (+)-C | 0.043 | 0.097 | 0.439 | 0.662 | 0.471 |
| k__Bacteria; p__Proteobacteria; c__Gammaproteobacteria; o__Enterobacteriales; f__Enterobacteriaceae; g__Pantoea; s__agglomerans | (+)-C | -5.138 | 12.635 | -0.407 | 0.686 | 0.471 |
| k__Bacteria; p__Proteobacteria; c__Gammaproteobacteria; o__Enterobacteriales; f__Enterobacteriaceae; g__; s__ | (+)-C | -0.171 | 0.428 | -0.399 | 0.691 | 0.471 |
| k__Bacteria; p__Proteobacteria; c__Gammaproteobacteria; o__Enterobacteriales; f__Enterobacteriaceae; g__; s__ | (+)-C | -2.042 | 5.382 | -0.379 | 0.706 | 0.474 |
| k__Bacteria; p__Proteobacteria; c__Gammaproteobacteria; o__Enterobacteriales; f__Enterobacteriaceae; g__; s__ | (+)-C | -0.071 | 0.445 | -0.160 | 0.874 | 0.518 |

**Supplementary Table S6. Association of OTUs composition diferences with plasma phenolic acids’ concentration differences in SD+ABX+BDPP and SD+BDPP groups.** Pair-wise correlations in ABX SD+BDPP and SD+BDPP of 62 indevisual OTUs’ proportion, identified in fecal samples collected at day 21 and BDPP-driven phenolic acids’ concentration of the 9 phenolic acids found to associate with memory preformance, analysed in plasma collected at day 22. Significant differences (p<0.01 at FDR≤0.107) are highlighted in red. (-), negative association.
